# A low-frequency *IL4R* locus variant in Japanese patients with intravenous immunoglobulin therapy-unresponsive Kawasaki disease

**DOI:** 10.1186/s12969-019-0337-2

**Published:** 2019-07-03

**Authors:** Yuji Amano, Yohei Akazawa, Jun Yasuda, Kazuhisa Yoshino, Katsuhiko Kojima, Norimoto Kobayashi, Satoshi Matsuzaki, Masao Nagasaki, Yosuke Kawai, Naoko Minegishi, Noriko Ishida, Noriko Motoki, Akira Hachiya, Yozo Nakazawa, Masayuki Yamamoto, Kenichi Koike, Toshikazu Takeshita

**Affiliations:** 1 Department of Microbiology and Immunology, Shinshu University School of Medicine, 3-1-1 Asahi, Matsumoto, Nagano 390-8621 Japan; 2 Department of Pediatrics, Shinshu University School of Medicine, 3-1-1 Asahi, Matsumoto, Nagano 390-8621 Japan; 3 Tohoku Medical Megabank Organization, Tohoku University, 2-1 Seiryo-machi, Aoba-ku, Sendai, Miyagi 980-8575 Japan; 4 Graduate School of Medicine, Tohoku University, 2-1 Seiryo-machi, Aoba-ku, Sendai, Miyagi 980-8575 Japan; 5 Graduate School of Information Science, Tohoku University, 6-3-09, Aramaki Aza-Aoba, Aoba-ku, Sendai, Miyagi 980-8579 Japan; 6 Shinonoi General Hospital, Minami Nagano Center, 666-1 Shinonoi, Nagano City, Nagano 388-8004 Japan

**Keywords:** Kawasaki disease, Interleukin-4 receptor, IVIG-resistance, Single nucleotide variant

## Abstract

**Background:**

Kawasaki disease (KD) is a systemic vasculitis which may be associated with coronary artery aneurysms. A notable risk factor for the development of coronary artery aneurysms is resistance to intravenous immunoglobulin (IVIG) therapy, which comprises standard treatment for the acute phase of KD. The cause of IVIG resistance in KD is largely unknown; however, the contribution of genetic factors, especially variants in immune-related genes, has been suspected.

**Methods:**

To explore genetic variants related to IVIG-unresponsiveness, we designated KD patients who did not respond to both first and second courses of IVIG therapy as IVIG-unresponsive patients. Using genomic DNA from 30 IVIG-unresponsive KD patients, we performed pooled genome sequencing targeting 39 immune-related cytokine receptor genes.

**Results:**

The single nucleotide variant (SNV), rs563535954 (located in the IL4R locus), was concentrated in IVIG-unresponsive KD patients. Individual genotyping showed that the minor allele of rs563535954 was present in 4/33 patients with IVIG-unresponsive KD, compared with 20/1063 individuals in the Japanese genome variation database (odds ratio = 7.19, 95% confidence interval 2.43–21.47). Furthermore, the minor allele of rs563535954 was absent in 42 KD patients who responded to IVIG treatment (*P* = 0.0337), indicating that a low-frequency variant, rs563535954, is associated with IVIG-unresponsiveness in KD patients. Although rs563535954 is located in the 3′-untranslated region of IL4R, there was no alternation in IL4R expression associated with the mior allele of rs563535954. However, IVIG-unresponsive patients that exhibited the minor allele of rs563535954 tended to be classified into the low-risk group (based on previously reported risk scores) for prediction of IVIG-resistance. Therefore, IVIG-unresponsiveness associated with the minor allele of rs563535954 might differ from IVIG-unresponsiveness associated with previous risk factors used to evaluate IVIG-unresponsiveness in KD.

**Conclusion:**

These findings suggest that the SNV rs563535954 could serve as a predictive indicator of IVIG-unresponsiveness, thereby improving the sensitivity of risk scoring systems, and may aid in prevention of coronary artery lesions in KD patients.

**Electronic supplementary material:**

The online version of this article (10.1186/s12969-019-0337-2) contains supplementary material, which is available to authorized users.

## Background

Kawasaki disease (KD), reported as paediatric acute mucocutaneous lymph node syndrome in 1967 [1], is now recognized as the most common cause of acquired heart disease among children in industrialized countries. While many researchers have investigated the role of infectious agents in the induction of KD, a specific infectious agent consistently detected in KD patients has not been identified. Epidemiological studies suggest that an ethnicity-dependent gene background is involved in KD. The incidences of KD in Japan and United States are 222.9 and 20.8, respectively, per 100,000 children < 5 years of age [2, 3]; similarly, the incidences for Japanese-American and White-American children in Hawaii are 210.5 and 13.7, respectively, per 100,000 children [4].

Although the development of coronary artery aneurysms has been observed in 20–25% of untreated KD patients [5, 6], treatment with intravenous immunoglobulin (IVIG) plus aspirin within the first 10 days of fever onset prevented coronary artery abnormalities [7–9]. Previous studies reported that 10–20% of KD patients were unresponsive to initial treatment with IVIG [10–13]; moreover, 20–30% of patients unresponsive to initial treatment with IVIG did not respond to a second dose of IVIG [10, 11]. Indeed, recent studies of refractory KD in Japan and Korea reported that 147/621 patients (23.7%) and 71/588 patients (12.1%), respectively, were unresponsive to initial IVIG therapy; of the unresponsive patients, 48 (7.7% of the total patients) and nine (1.5% of the total patients), respectively, remained resistant to a second dose of IVIG [14, 15]. In addition, 9/48 patients (18.8%) unresponsive to a second dose of IVIG developed coronary artery abnormalities, indicating a high incidence of coronary artery abnormalities in IVIG-unresponsive KD patients and the need for prediction of IVIG-unresponsiveness. Here, we screened single nucleotide variants (SNVs) as potential risk factors in KD patients resistant to a second dose of IVIG and found that a low-frequency variant located in the *IL4R* locus was associated with IVIG-unresponsiveness in KD patients.

## Methods

### Subjects

From 2012 to 2016, KD patients (typical KD (*n* = 75), atypical KD (*n* = 7)) and allergic subjects (*n* = 99) were enrolled in this study at Shinshu University Hospital or its affiliated hospitals, after parental informed consent. Typical KD patients fulfilled the diagnostic criteria for typical KD (patients with five or six principal symptoms, or patients with four principal symptoms *and* coronary artery lesions [CAL]) [16], and were treated with IVIG and aspirin as first-line treatment within 8 days of fever onset. Typical KD patients who failed to respond to initial therapy with IVIG were re-treated with IVIG and aspirin. Typical KD patients who failed to respond to both the first and second courses of IVIG, and were treated with anti-TNFα antibody, plasma exchange, or both, were diagnosed with IVIG-unresponsive KD (*n* = 33). Typical KD patients who responded within two courses of IVIG were defined as IVIG-responsive KD (*n* = 42). Unresponsiveness to IVIG therapy was assessed by the presence of an axial temperature of > 37.5 °C that persisted for > 24 h after IVIG treatment. KD patients who presented with three principal symptoms, with or without CAL, or four principal symptoms without CAL, were defined as atypical KD (*n* = 7). Paediatric patients who were diagnosed with allergic disease (atopic dermatitis, bronchial asthma, or food allergy) and showed no history of KD were used as non-KD subjects. This study was approved by the Institutional Review Board of Shinshu University School of Medicine. The project protocol was reviewed and approved by the Ethnic Committee of Tohoku University Graduate School of Medicine and by the Ethics Committee of Iwate Medical University. All methods were performed in accordance with the relevant guidelines and regulations.

### Pooled genomic DNA sequencing

Genomic DNA was extracted from white blood cells with a standard procedure and quantified by measuring the absorbance at 260 nm. Each 500 ng of genomic DNA from 30 IVIG-unresponsive KD patients were pooled and used to prepare next-generation sequencing libraries. Candidate genetic regions were individually amplified with specific primers (Additional file 1: Table S1, S2), KOD FX neo DNA polymerase (Toyobo, Osaka, Japan), and 200 ng of pooled genome as a template. Amplified products were purified with Agencourt AMPure XP (Beckman Coulter, Brea, CA, USA) and shearing was performed by sonication. Fragmented amplicons were individually separated by agarose gel electrophoresis and 230–250 bp fragments were collected. Sequencing templates were prepared with the Ion PGM Template OT2 Reagents 200 Kit (Thermo Fisher, Waltham, MA, USA), and next-generation sequencing was performed by Ion PGM with a 318 chip (Thermo Fisher), according to the Ion torrent protocol. Base calling, reference mapping, and variant calling were performed with Torrent Suite Software. Allele frequencies of detected variants were compared with 1KGP and 1KJPN.

### Genotyping and statistical analysis

To confirm the precise allele frequencies of rs563535954, rs193167358, and rs538765536, which were screened by next-generation sequencing, the target genomic region was amplified individually with specific primers (Additional file 1: Table S1) and KOD plus neo DNA polymerase. Amplicons were sequenced with Sanger Technology (Applied Biosystems, Foster City, CA, USA), and sequencing data were analysed with Chromas software (Technelysium Pty Ltd., South Brisbane, Australia). Statistically significant differences were determined by Fisher’s exact test (two-sided *P* <  0.05).

### Allele-specific quantification of *IL4R* transcripts

Peripheral blood mononuclear cells from KD patients were obtained by density gradient centrifugation with Lymphocyte Separation Medium (ICN Biomedicals Inc., Santa Ana, CA, USA). Cells were stimulated with 5 μg/mL PHA and 1 nM IL-2 for 2 days, and total RNA was isolated with the ISOGEN II reagent (Wako, Tokyo, Japan). Then, 1 μg of total RNA was used for the oligo (dT20)-primed synthesis of cDNA with Superscript III reverse transcriptase (Invitrogen, Carlsbad, CA, USA). To quantify the relative abundance of the two alleles of the *IL4R* transcript, we used a Hot-stop PCR technique [17]. Short fragments containing the rs563535954 region were individually amplified with cDNA or genomic DNA, respectively, as a template. At the last cycle of PCR, we added an Alexa488-labelled reverse primer to discern homoduplexes. After purification and quantification, amplicons were digested with *Bam*HI, followed by separation with polyacrylamide gel electrophoresis. Alexa488-labelled fragments were detected by a Typhoon trio imager (GE healthcare, Little Chalfont, UK), and quantification was performed with ImageJ software. Primers used in this assay are described in Additional file 1: Table S1.

### Luciferase assay

To form the WT construct, the 3′-UTR and 5′-UTR of human *IL4R* transcript variant 3 were cloned into a pmirGLO Dual Luciferase expression vector (Promega, Madison, WI, USA) at the 3′-end and 5′-end of firefly luciferase, respectively. Constructs containing the minor allele of rs563535954 were generated with an inverse PCR method. Then, 200 ng of each plasmid was transfected into HEK293T cells (1 × 10^5^) with 1 μg of polyethylenimine MAX (Polysciences Inc., Warrington, PA, USA). After incubation for 24 h, luciferase expression was monitored with a Dual-Glo Luciferase Assay System (Promega). To evaluate the effects of miRNAs, 100 ng of luciferase plasmid and 100 pmol of each miRNA mimic (Bioneer, Daejeon, Korea) were co-transfected into HeLa cells (1 × 10^5^) with 2 μg of polyethylenimine MAX; luciferase activity was measured 24 h after transfection.

### Epstein-Barr virus-transformed B lymphoblastoid cell line (B-LCL)

B-LCLs established from healthy donors with heterozygous rs563535954 were obtained from Tohoku Medical Megabank Organization. B-LCLs were maintained in RPMI-1640 medium supplemented with 10% foetal bovine serum, 40 U/ml penicillin, and 100 μg/ml streptomycin. To perform the allele-specific quantification of *IL4R* transcripts described above, cells were incubated with 5 μg/ml Actinomycin D (Nacalai Tesque, Kyoto, Japan), 10 ng/ml IL-4, 10 ng/ml TNFα, or 10 ng/ml IFNγ (PeproTech, Rocky Hill, NJ, USA) for the indicated times.

## Results

### Pooled genome sequencing of cytokine receptors in IVIG-unresponsive KD patients

**Fig. 1:**
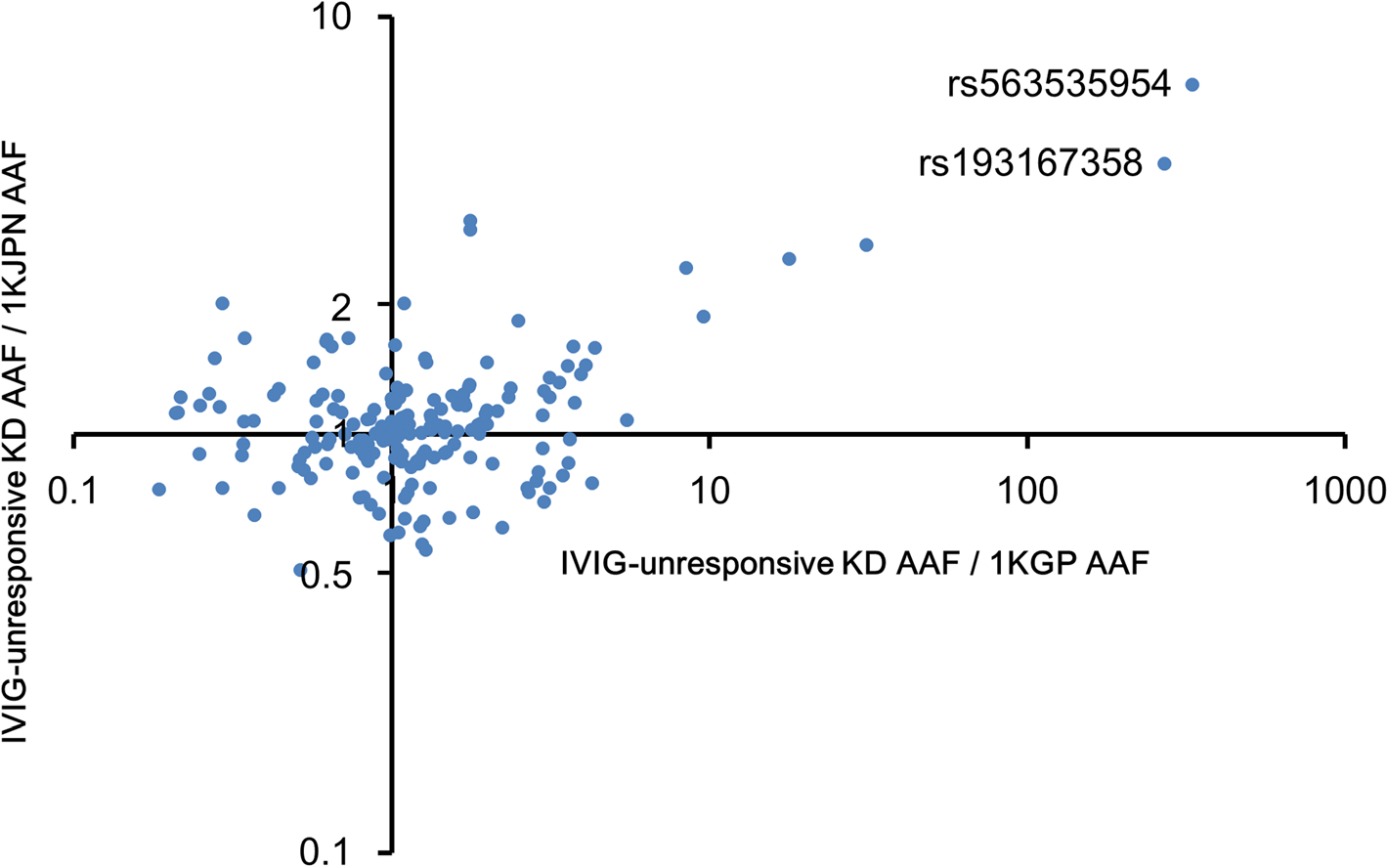
Comparison of the frequency of SNVs in cytokine receptors between IVIG-unresponsive KD patients and reference individuals in whole-genome sequencing projects. Plots indicate 193 SNVs, which were detected in the gene loci of cytokine receptors (alternative allele frequency: AAF > 0.05) in 30 unresponsive KD patients. Vertical axis: the values obtained by dividing the AAF of 193 SNVs estimated through pooled genome sequencing of 30 KD patients by the AAF of that found in the 1KJPN. Horizontal axis: the values obtained by dividing the AAF of 193 SNVs estimated through the pooled genome sequencing by the AAF of that found in the 1KGP

Genome-wide association studies of KD extracted several susceptibility gene loci related to KD; single nucleotide polymorphisms (SNPs) in the loci were identified as risk factors that affected KD. Notably, most of these SNPs were located in or near immune-related genes [18–24]. We investigated genetic variants of cytokine receptors, which are closely involved in various immune responses. Because the final exons of cytokine receptors include a regulatory region for gene expression such as 3′-UTR, we selected the final exons of 39 cytokine receptors and eight splicing forms as the analysis region (Additional file 1: Table S2). We pooled the genomes of 30 KD patients who received treatment with IVIG plus aspirin within the first 8 days of fever onset and a second IVIG therapy (because of the absence of a response to initial treatment), but who remained unresponsive to the second treatment. We performed next-generation sequencing and found 206 SNVs and six insertions/deletions (indels) (read depth > 450, detected frequency > 0.05) (Additional file 1: Table S3). We found that 193 of the 206 SNVs and zero of the six indels were present in a reference panel of 1070 Japanese individuals catalogued by the 1000 genome project of the Tohoku Medical Megabank Organization (1KJPN) [25]. The frequencies of the 193 SNVs observed in 30 KD patients correlated with the frequencies of the same 193 SNVs in 1KJPN (Additional file 1: Figure S1, *R*^2^ = 0.9331). The allele frequencies of the 193 SNVs in the 30 patients were compared with their frequencies in 1KJPN and 2504 individuals from the 1000 genome project database (1KGP) comprising several ethnic groups [26]. We found that the minor allele of rs563535954 located in the 3′-UTR of the interleukin-4 receptor α (IL-4Rα) gene, *IL4R*, was concentrated in IVIG-unresponsive KD patients (Fig. 1). The minor allele of rs563535954 was present in 20/1063 individuals from 1KJPN, in four of 33 patients in the current study who were unresponsive to IVIG therapy and who were individually analysed by genotyping (OR = 7.19, 95% CI: 2.43–21.47; comparison with 1KJPN, Table 1), and in one of 2504 individuals from 1KGP. Of these 2504 individuals, 2400 were not Japanese and did not harbour rs563535954; of the remaining 104 Japanese individuals, one had the minor allele. The 20 1KJPN individuals and four patients described above were heterozygous rs563535954 (C/T), but not homozygous rs563535954 (T/T). Although another SNV, rs193167358, was estimated in four patients by pooled genome sequencing, heterozygous rs193167358 was detected in only two patients by individual genotyping (Fig. 1 and Table 1).

**Table 1:** **Table 1** Individual genotyping results of rs563535954 and rs193167358 in 33 KD patients unresponsive to IVIG therapy

	IVIG-unresponsive KD		1KJPN		OR(95%CI)	*P* value
	−/+	−/−	−/+	−/−		
rs563535954	4	29	20	1043	7.193(2.430–21.474)	0.0047
rs193167358	2	31	26	1044	2.591(0.656–10.322)	0.203

Individual genotyping results of rs563535954 and rs193167358 in 33 KD patients unresponsive to IVIG therapy. *P* values were obtained by comparison between IVIG-unresponsive KD patients and individuals from 1KJPN, a Japanese genome reference panel, using Fisher’s exact test (two-sided). (+) denotes number of subjects with the minor allele.

### Genotyping of rs563535954 in IVIG-responsive KD patients

We investigated the frequency of rs563535954 in IVIG-responsive KD patients who received treatment with IVIG plus aspirin within the first 8 days of fever onset, which resulted in improvements of fever and acute inflammatory markers within two courses of treatment. None of the 42 IVIG-responsive cases had the minor allele of rs563535954. Consequently, rs563535954 was considered to be associated with IVIG-unresponsiveness, rather than the onset of KD (*P* = 0.0337, Table 2). In addition, we investigated whether the minor allele of rs563535954 was concentrated in the population of this study region. However, rs563535954 was not present in any of 99 children with allergy, a common paediatric disease, who had no history of KD but exhibited a similar background (gender ratio and living place) to that of the KD patients.

**Table 2:** **Table 2** **a** Characteristics of typical KD patients responsive to IVIG therapy ( n = 42) and those unresponsive to a second dose of IVIG therapy ( n = 33). **b** Prevalence of the minor allele of rs563535954 in IVIG-unresponsive and IVIGresponsive KD patients

A					
	Patients with typical KD (*n* = 75)				
	IVIG responders	IVIG non-responders			
	(*n* = 42)	(*n* = 33)			
Age at diagnosis (month)					
Mean (SD)	36.1 (21.9)	31.6 (18.3)			
Median (range)	33 (4–121)	30 (4–81)			
Gender					
Male / Female	1.3	2.3			
Principal symptoms ± CAL (%)					
4 symptoms + CAL	1 (2.4%)	3 (9.1%)			
5 or 6 symptoms - CAL	34 (81.0%)	16 (48.5%)			
5 or 6 symptoms + CAL	8 (16.7%)	14 (42.4%)			
B					
	(*n* = 33)		(*n* = 42)		*P* value
	IVIG-unresponsive KD		IVIG-responsive KD		
	C/T	C/C	C/T	C/C	
rs563535954	4	29	0	42	0.0337

Association of rs563535954 with IVIG responsiveness in KD patients. (A) Characteristics of typical KD patients responsive to IVIG therapy (*n* = 42) and those unresponsive to a second dose of IVIG therapy (*n* = 33). Coronary artery lesions (CAL) include ectasia and aneurysm. (B) Prevalence of the minor allele of rs563535954 in IVIG-unresponsive and IVIG-responsive KD patients. *P* value was obtained by comparison between IVIG-unresponsive KD patients (*n* = 33) and IVIG-responsive KD patients (*n* = 42), using Fisher’s exact test (two-sided). (T) denotes presence of the minor allele.

### Influence of rs563535954 on the stability and translation of IL-4Rα mRNA

The 3′-UTR region of genes is known to affect the stability and translation of mRNA. Therefore, we compared the amounts of IL-4Rα mRNA derived from the minor allele (T) with the counterpart allele (C) of rs563535954. Using mRNA extracted from phytohaemagglutinin (PHA)-stimulated peripheral blood lymphocytes of three KD patients with heterozygous rs563535954 (C/T), we performed allele-specific transcript quantification (ASTQ) (Additional file 1: Figure S2A). The ASTQ, *Bam*HI digested, Alexa-488 labelled homoduplex PCR products (Digested PCR products) showed that the ratio between products from the minor and counterpart alleles was nearly equal (Additional file 1: Figure S2B). Next, we established three Epstein-Barr virus (EBV)-transformed B lymphoid cell lines (B-LCLs) with heterozygous rs563535954 (C/T) derived from volunteers of 1KJPN; these were used to investigate the stability of IL-4Rα mRNA derived from each allele. The B-LCLs were incubated with actinomycin D and harvested at the indicated times. No difference was found between digested PCR products derived from each allele (Additional file 1: Figure S3A). Therefore, we investigated the quantitative ratio of IL-4Rα mRNA derived from each allele in B-LCLs under cytokine stimulation. The ratio of digested PCR products derived from the minor allele of rs563535954 (T) and the counterpart allele (C) were similar, despite stimulation with the cytokines IL-4, TNF-α, or IFN-γ (Additional file 1: Figure S3B). Because we did not detect a difference in IL-4Rα mRNA expression or stability derived from each allele under cytokine stimulation or in steady-state conditions, we investigated the effect of rs563535954 on translation efficiency with a 3′-UTR-inserted dual luciferase reporter vector (Additional file 1: Figure S4A). There was no difference in firefly-luciferase expression between the 3′-UTR-inserted dual luciferase reporter vector with the minor allele (T) and the same vector with the counterpart allele (C) (Additional file 1: Figure S4B). MicroRNAs (miRNAs) serve as post-transcriptional regulators of mRNAs, through regulation of the stability of the 3'-UTR of target mRNAs. We thus speculated that translational control of the 3'-UTR of IL-4Rα might be altered, with or without the minor allele of rs563535954, in the presence of miRNAs that exhibit the potential to bind to the rs563535954 region of the 3′-UTR. To identify candidate miRNAs, we used microRNA.org (http://www.microrna.org/microrna/home.do) and TargetScan (www.targetscan.org); we identified four miRNAs (miR-615-5p, miR-663a, miR1228-5p, and miR1908-5p). By using 3′-UTR-inserted dual luciferase reporter vectors, we measured translation efficiency in the presence of each miRNA mimic described above. We did not observe any differences among any of the conditions (Additional file 1: Figure S5).

### Re-sequencing analysis of the *IL4R* locus in IVIG-unresponsive patients

**Fig. 2:**
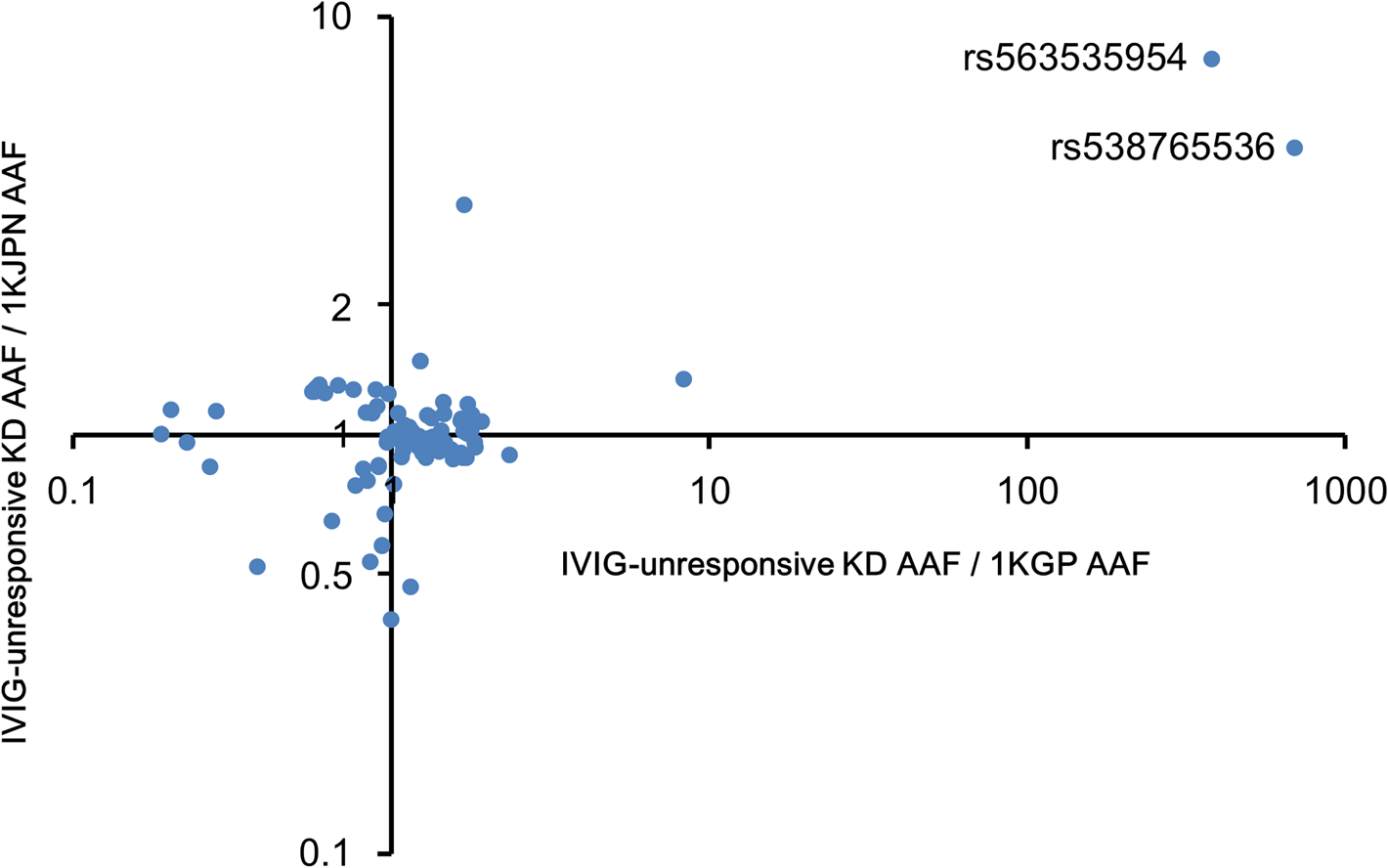
Comparison of the frequency of SNVs in the *IL4R* locus between IVIG-unresponsive KD patients and reference individuals in whole-genome sequencing projects. Plots indicate 99 SNVs in the *IL4R* locus (58 kbp including the LD block with rs563535954) (AAF > 0.05) detected in 30 unresponsive KD patients. Vertical axis: the values obtained by dividing the AAF of 99 SNVs estimated through pooled genome sequencing of 30 KD patients by the AAF of that found in the 1KJPN. Horizontal axis: the values obtained by dividing the AAF of 99 SNVs estimated through the pooled genome sequencing by the AAF of that found in the 1KGP

Because another SNV genetically linked to rs563535954 might exist and be substantively associated with second treatment-unresponsive KD patients, we focused on the genome area around rs563535954, an approximately 3.7-kb linkage disequilibrium (LD) block (Additional file 1: Figure S6 (high-resolution image was shown in Additional file 2)). We sequenced the regions of the 3.7-kb LD block and full-length *IL4R* locus in 30 KD patients resistant to a second dose of IVIG therapy, which revealed 108 SNVs and 11 indels (read depth > 1200, detected frequency > 0.05) (Additional file 1: Table S4). Of these, 99 SNVs and zero indels were present in the 1KJPN (Additional file 1: Figure S7, *R*^2^ = 0.9363). A novel SNV, rs538765536, which might be related to second treatment-unresponsiveness in KD patients, was detected (Fig. 2). The minor allele of rs538765536, present in 58 individuals of the 1KJPN and located near the 3.7-kb LD block (Additional file 1: Figure S6), was detected in five of 33 KD patients unresponsive to second treatment (OR = 3.12, 95% CI: 1.20–8.11; comparison with 1KJPN). Fifty-eight individuals in the 1KJPN and the five patients described above exhibited heterozygous rs538765536. Because four of these five KD patients exhibited both rs538765536 and rs563535954, we investigated the relationship between rs538765536 and rs563535954 in the 1KJPN. Seventeen of 58 (29.3%) individuals with rs538765536 had rs563535954, and 17 of 20 (85.0%) individuals with rs563535954 had rs538765536; this indicated that these alleles were genetically linked. Moreover, four (80%) of five KD patients with rs538765536 also had rs563535954, although 29% of individuals with rs538765536 had rs563535954 in 1KJPN. This observation suggested that the minor allele of rs538765536 is not related to IVIG-unresponsiveness in KD patients, independent of rs563535954. However, four of 33 patients had rs563535954 and rs538765536, suggesting that this haplotype is closely related to second treatment-unresponsiveness in KD patients.

### Usability of rs563535954 for prediction of IVIG-resistance in KD patients

**Fig. 3:**
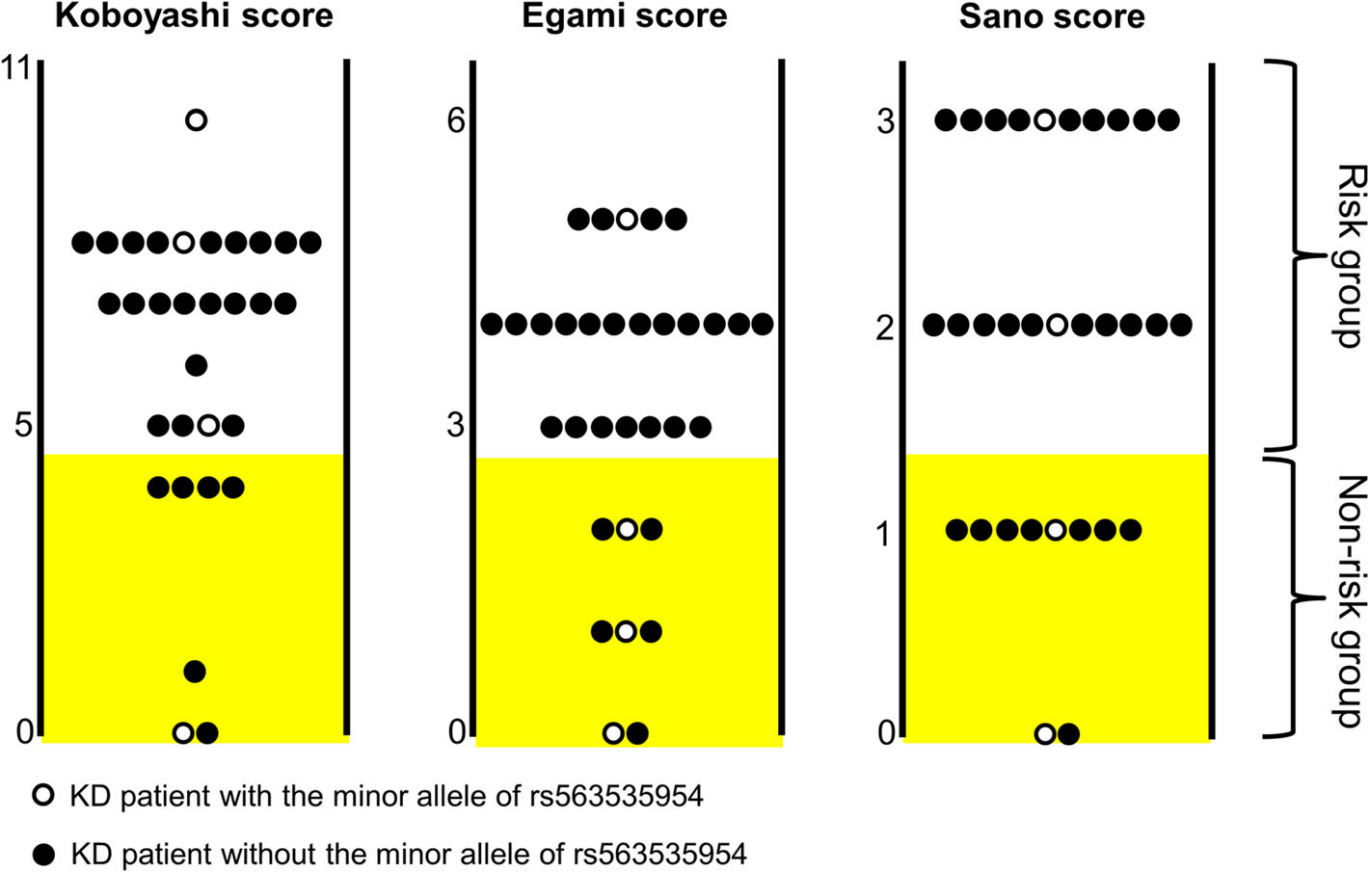
Evaluations using the three risk scoring systems in KD patients unresponsive to a second dose of IVIG. Kobayashi, Egami, and Sano risk scores are shown for 31, 32, and 33 IVIG-unresponsive patients. Highlighted zones indicate non-risk groups in each risk scoring system

Prediction of IVIG-unresponsiveness during early onset of KD may help to prevent the development of coronary artery lesions (CAL). We considered the possibility that rs563535954 could serve as a novel risk index of IVIG-unresponsiveness. Therefore, we assessed Kobayashi, Egami, and Sano scores, the most common risk scoring systems related to IVIG-unresponsiveness, in our IVIG-unresponsive subjects [27–29]. Kobayashi, Egami, and Sano scores have been reported to predict IVIG-unresponsiveness with sensitivity = 76% (specificity = 80%), 78% (76%) and 77% (86%), respectively. Of 33 KD patients unresponsive to second treatment, clinical data of 31, 32, and 31 patients contained sufficient data for evaluation with Kobayashi, Egami, and Sano scores, respectively [27–29]. In the IVIG-unresponsive KD patients who did not exhibit the minor allele of rs563535954, these risk scores predicted IVIG-unresponsiveness with sensitivity of approximately 70–80% (Fig. 3). In contrast, only 25 and 50% of IVIG-unresponsive KD patients who exhibited the minor allele of rs563535954 were predicted by Egami and Sano scores, respectively (Fig. 3). Particularly with respect to the Egami score, a greater proportion of IVIG-unresponsive KD patients who exhibited the minor allele of rs563535954 were classified into the non-risk group (Table 3). As mentioned above, the minor allele of rs563535954 was not observed in 42 IVIG-responsive KD patients. These results suggest that rs563535954 could be a risk index independent of clinical features.

**Table 3:** **Table 3** Differences of IVIG-resistance risk assessment between patients with or without rs563535954

Risk score	IVIG-unresponsive Patients				OR(95%CI)	*P* value
	rs563535954 −/+		rs563535954 −/−			
	Risk	Non-risk	Risk	Non-risk		
Kobayashi	3	1	21	6	1.167(0.102–13.363)	1.000
Egami	1	3	23	5	13.800(1.178–161.719)	0.0393
Sano	2	2	19	8	2.375(0.283–19.925)	0.3871

Differences of IVIG-resistance risk assessment between patients with or without rs563535954. *P* values were obtained by comparison between IVIG-unresponsive KD patients with and without the minor allele of rs563535954, using Fisher’s exact test (two-sided). (+) denotes presence of the minor allele.

## Discussion

Previous studies reported that 10–20% of KD patients unresponsive to initial treatment with IVIG showed an increased risk for the development of coronary artery aneurysms [10–13]. The origin of unresponsiveness to IVIG therapy and the mechanism of action of IVIG therapy both remain unknown; therefore, it is important to elucidate the mechanisms involved to prevent the development of coronary artery aneurysms. Although the number of patients unresponsive to a second dose of IVIG is small (1.5–7.7% of total KD patients) [14, 15], the analysis of genetic variants in unresponsive patients might facilitate elucidation of the IVIG-unresponsive mechanism or risk factors. Here, we showed that rs563535954 identified in Japanese KD patients was a low-frequency variant located in the *IL4R* locus, and that it was concentrated in KD patients unresponsive to a second dose of IVIG. We investigated the effect of the minor allele of rs563535954 on gene expression, because rs563535954 is located in the 3′-UTR of *IL4R*. However, we did not distinguish a difference in either mRNA stability or translation efficiency between a reference allele and the minor allele of rs563535954. The gene expression level of the minor allele might be influenced by a specific environment, including inflammatory factors as described below.

Whether IL-4 levels are increased in KD patients is controversial [30–32]. IL-4 was recently reported to act as a proinflammatory cytokine against vascular endothelial cells [33–36], leading to vascular leakage and endothelial barrier dysfunction. Accordingly, the expression of IL-4Rα derived from the minor allele might be affected by a specific environment accompanied by inflammatory events. Therefore, we used EBV-transformed B cell lines expressing heterozygous rs563535954 (C/T) to compare transcription from each allele under stimulation with IL-4, TNF-α, or IFN-γ; however, no effect was observed upon stimulation with any of the cytokines (Additional file 1: Figure S3B). Contrasting evidence regarding the effect of IL-4 has been reported in a mouse model of arthritis. While IVIG suppressed arthritis, arthritic inflammation in mice lacking IL-4 was not suppressed by IVIG [37], indicating that IL-4 was required for the therapeutic effect of IVIG. These data indicate that, IL-4 is a key molecule in inflammatory responses. Further studies of inflammatory environments are required to explore the effect of the minor allele of rs563535954.

IL-4 and IL-4Rα are involved in allergic disease, and KD patients have been reported to exhibit an atopic trend [38–40]. In addition, SNPs in the gene locus of IL-4Rα have been associated with allergic disease [41–43]. Based on this background, we genotyped rs563535954 in 99 children with allergy as non-KD subjects; however, no individuals had the minor allele of rs563535954, suggesting that rs563535954 is not a risk variant of allergic disease.

To clearly distinguish between IVIG-responsive KD patients and IVIG-unresponsive KD patients, we performed a study of typical KD patients with at least five of the clinical features of KD, or four of the clinical features plus CAL (including ectasia and aneurysm) [16]. We further examined atypical KD patients because the formation of CAL, which is a trigger for severe complications of KD, is also observed in atypical KD patients. We genotyped seven patients with atypical KD and found that one had the minor allele of rs563535954. The patient demonstrated only three of the clinical features of KD; therefore, diagnosis was delayed. IVIG treatment of the patient was initiated at day 14 after fever onset and the effect of the treatment was not defined because of long-term treatment with IVIG for 5 days. Of note, the patient presented with a giant aneurysm (internal diameter > 8 mm) as serious KD. Therefore, regardless of IVIG-responsiveness, we re-examined 82 KD patients, including atypical KD patients, based on the formation of CAL (Table 4A). In 82 KD patients, including seven who received IVIG therapy as atypical KD with three or four of the clinical features, 26 patients had CAL; four of these 26 had the minor allele of rs563535954. Of 56 patients without CAL, only one had the minor allele of rs563535954. Accordingly, the formation of CAL was frequently observed in patients with heterozygous rs563535954, compared with patients without rs563535954 (OR = 10.00, 95% CI: 1.39–69.52; *P* = 0.0331, Table 4B). These findings prompted us to consider the possibility that the minor allele of rs563535954 may be associated with the progress of vasculitis in KD.

**Table 4:** **Table 4** **a** Characteristics of KD patients with ( n = 26) or without (n = 56) CAL formation. **b** Prevalence of the minor allele of rs563535954 in KD patients with and without CAL formation

A						
	All KD patients (*n* = 82)					
	Without CAL formation (*n* = 56)	With CAL formation (*n* = 26)				
Age at diagnosis (month)						
Mean (SD)	30.7 (16.8)	39.8 (26.3)				
Median (range)	30 (4–67)	35 (10–121)				
Gender						
Male / Female	1.2	4.2				
Principal symptoms (%)						
3 symptoms	2 (3.6%)	1 (3.8%)				
4 symptoms	4 (7.1%)	4 (15.4%)				
5 or 6 symptoms	50 (89.3%)	21 (80.8%)				
B						
	KD patients					
	+ CAL (*n* = 26)		- CAL(*n* = 56)			
rs563535954	T/C	C/C	T/C	C/C	OR(95%CI)	*P* value
	4	22	1	55	10.000(1.392–69.521)	0.0331

Association of rs563535954 with CAL formation in KD patients. (A) Characteristics of KD patients with (*n* = 26) or without (*n* = 56) CAL formation. (B) Prevalence of the minor allele of rs563535954 in KD patients with and without CAL formation. *P* value was obtained by comparison between KD patients with and without CAL formation using Fisher’s exact test (two-sided). (T) denotes presence of the minor allele.

Prediction of IVIG-unresponsiveness in the early phase of KD onset is an important aspect in preventing development of CAL. Several risk scoring systems of IVIG-unresponsiveness that utilize clinical features detect most IVIG-unresponsive individuals and contribute to therapeutic decisions for KD patients [27–29]. However, approximately 20% of IVIG-unresponsive patients are classified into non-risk groups by these risk scoring systems. A portion of these patients may exhibit genetic predispositions to IVIG-unresponsiveness and resistance to IVIG therapy, despite lacking clinical features of IVIG-unresponsiveness. We observed that IVIG-unresponsive KD patients with the minor allele of rs563535954 tended to be classified into the non-risk group. Particularly with respect to the Egami score, three of four IVIG-unresponsive patients with the minor allele of rs563535954 were classified into the non-risk group, whereas five of 28 IVIG-unresponsive patients without the minor allele of rs563535954 were classified into the non-risk group (Table 3). This result supports our conclusion that the minor allele of rs563535954 contributes to the genetic predisposition associated with IVIG-unresponsiveness in KD patients.

We focused on KD patients who were unresponsive to a second dose of IVIG in this study, and our sample size was small. There are some treatment approaches available for KD patients who are unresponsive to the initial dose of IVIG. While there is an increased proportion of patients who undergo combined therapy with steroid, infliximab (anti-TNFα antibody), or immunosuppressant as second or later line therapy, IVIG remains the standard therapy for KD. Our results consistently suggest an association between rs563535954, a genetic variant in the 3′-UTR of *IL4R*, and IVIG-unresponsiveness in KD patents. Nevertheless, our sample size was small; therefore, further validation should be performed in additional cohorts with greater numbers of patients.

## Conclusions

We identified a low-frequency SNV, rs563535954, associated with IVIG-unresponsiveness in Japanese KD patients (*P* = 0.0337). We also observed the association between the minor allele of rs563535954 and CAL formation in KD patients (*P* = 0.0331). Our findings might contribute to the prediction of IVIG-unresponsiveness and CAL formation in KD patients, and may provide a biological basis for the development of a therapy for IVIG-unresponsive KD.

## Additional files

Additional file 1: **Table S1.** List of primers used in this study. **Table S2.** A list of 39 cytokine receptors and the 8 splice forms screened by pooled genome sequencing. **Table S3.** A list of 206 SNVs and 8 Indels in the gene loci of cytokine receptors detected in 30 IVIG-unresponsive KD patients. **Table S4.** A list of 108 SNVs and 11 indels in the *IL4R* locus including the LD block with rs563535954 detected in 30 IVIG-unresponsive KD patients. **Figure S1.** Pairwise correlation between AAFs of SNVs, estimated via pooled genome sequencing of IVIG-unresponsive KD patients, in the gene loci of cytokine receptors and AAFs found in 1KJPN. **Figure S2.** Allele-specific transcript quantification of *IL4R* in PBMCs from IVIG-unresponsive KD patients with heterozygous rs563535954. **Figure S3.** Allele-specific transcript quantification of IL4R in B-LCLs derived from individuals with heterozygous rs563535954. **Figure S4.** Effect of the minor allele of rs563535954 on translational efficiency. **Figure S5.** The effect of miRNA mimics on the activity of luciferase expressed from a reporter plasmid containing the 3’-UTR of IL-4Rα. **Figure S6.** LD plot around the *IL4R* gene locus and *IL4R* gene structure. **Figure S7.** Pairwise correlation between the AAFs of SNVs, estimated via the pooled genome sequencing of IVIG-unresponsive KD patients, in the *IL4R* gene locus including the LD block with rs563535954 and AAFs of that found in 1KJPN. (PDF 2572 kb)

Additional file 2: High-resolution image of Additional file 1: Figure S6. (TIF 3930 kb)

## Data Availability

All data generated during this study are included in this published article and its supplementary information files.

## Abbreviations

ASTQ: Allele-specific transcript quantification
B-LCL: B lymphoblastoid cell line
CAL: Coronary artery lesions
EBV: Epstein-Barr virus
IVIG: Intravenous immunoglobulin
KD: Kawasaki disease
LD: Linkage disequilibrium
SNP: Single nucleotide polymorphism
SNV: Single nucleotide variant
