# Supplementary material for: A low-frequency IL4R locus variant in Japanese patients with intravenous immunoglobulin therapy-unresponsive Kawasaki disease
Source: Pediatr Rheumatol Online J. 2019 Jul 3;17:34. doi: 10.1186/s12969-019-0337-2 (PMC6610867; doi:10.1186/s12969-019-0337-2)
Supplement: Supplementary file 1 — Table S1. List of primers used in this study. Table S2. A list of 39 cytokine receptors and the 8 splice forms screened by pooled genome sequencing. Table S3. A list of 206 SNVs and 8 Indels in the gene loci of cytokine receptors detected in 30 IVIG-unresponsive KD patients. Table S4. A list of 108 SNVs and 11 indels in the IL4R locus including the LD block with rs563535954 detected in 30 IVIG-unresponsive KD patients. Figure S1. Pairwise correlation between AAFs of SNVs, estimated via pooled genome sequencing of IVIG-unresponsive KD patients, in the gene loci of cytokine receptors and AAFs found in 1KJPN. Figure S2. Allele-specific transcript quantification of IL4R in PBMCs from IVIG-unresponsive KD patients with heterozygous rs563535954. Figure S3. Allele-specific transcript quantification of IL4R in B-LCLs derived from individuals with heterozygous rs563535954. Figure S4. Effect of the minor allele of rs563535954 on translational efficiency. Figure S5. The effect of miRNA mimics on the activity of luciferase expressed from a reporter plasmid containing the 3’-UTR of IL-4Rα. Figure S6. LD plot around the IL4R gene locus and IL4R gene structure. Figure S7. Pairwise correlation between the AAFs of SNVs, estimated via the pooled genome sequencing of IVIG-unresponsive KD patients, in the IL4R gene locus including the LD block with rs563535954 and AAFs of that found in 1KJPN. (PDF 2572 kb) [file 12969_2019_337_MOESM1_ESM.pdf]

Supplemental Material

Primer sequence used for library construction for next generation sequencing in *IL4R* locus

Targeted region (GCRh38)	Primer sequence	Amplicon size(bp)
chr16:27308945-27318883	5'-CTAGAGTGGCTAAAATCCTCAAACTGACAATATCAAAAGC-3'<br>5'-TTTGCTTTGCAGGCGAGAGGCTCTAAGACC-3'	9939
chr16:27318605-27328663	5'-CTAGCTCTGACTGACTCATTCTCTCTTTAGGTGC-3'<br>5'-GAGCTTTATGTCTGCTGTGCTCACTGCTTTATCC;3'	10059
chr16:27328577-27339372	5'-TATGTTTATTGTACACATGCTATGCCTGTGTCAGGCAC-3'<br>5'-TCCATATAGGCTGCTATAACAAAAATACCTTAGAGCAGG-3'	10796
chr16:27339280-27349114	5'-AGATGCACACCATTGCACCCAATAGAGCAATACG-3'<br>5'-TCACTAAATTCTTTATGTTCAGCTCTGTTCTTGGGAAGG-3'	9835
chr16:27349009-27358894	5'-AGGCAGAAGAATGAAGTCCAGCTTCTCTTGTGG-3'<br>5'-TGTTGATCTCTGAATTATTGACAGGTGAACGCTCC-3'	9886
chr16:27358724-27366771	5'-ATCTGTAGAATAGGGTTAGCAATAGAATCCATGTCACC-3'<br>5'-GATAATCATTGAGCCTCCCAACTAGAGCTGTTTGG-3'	8048

Primer sequence used for individual genotyping by Sanger DNA sequencing

Target SNV	primer	application
rs563535954	5'-TAATTCTTGTAGAGATGTTTGAGACGGCTTGGGCTC-3'<br>5'-GGATTACTTCCTAAATCAACTATTTGAATCCTTGTATGG-3'	amplify the SNV region
	5'-TGTGCGGCCACCTGAAACAGTGTC-3'	sequencing
rs193167358	5'-TCAGGCAGCCAAAGGTGGAATAGTAACATCTGG-3'<br>5'-CATGTTTTTCAGGGAGGCAGTGAAGCTGTGG-3'	amplify the SNV region
	5'-TATTTATGTGACCTGGCAGATCTCTG-3'	sequencing
rs538765536	5'-AGAAGCGGTCCCGAGGCCAGGAACC-3'<br>5'-TGGAAAGGCATCTCTTTGGCAGCCTTGTGAGG-3'	amplify the SNV region
	5'-TTCTGGAGTATGGAAAGGTCCAGC-3'	sequencing

Primer sequence used for allele specific *IL4R* transcript quantification

5’-TGGAAGGCAGCCAGGCTGGCAGATTTC-3’
5’-CTCCCTGAAGTTCAATTTCTCATCGATTTCCCAAGG-3’

Supplementary Table 1. List of primers used in this study.

Supplementary Table 2. A list of 39 cytokine receptors and the 8 splice forms screened by pooled genome sequencing. Primers used for next generation sequencing library preparation are listed.

gene	Reference Sequence (mRNA)	Reference sequence Accession number	Chr	GCRh38 position	candidate exon	Fw Primer	Rv Primer	Amplicon size
TNFRSF1B	Homo sapiens TNF receptor superfamily member 1B (*TNFRSF1B*), mRNA	NCBI Reference Sequence: NM_001066.2	1	chr1:12206553-12209614	10	GAAAGAAGAGGATTATAGGGATCAGGGATGTCC	CACCCGGCCTCAACTATGATTAAAACAAACAAAC	1099
IL23R	Homo sapiens interleukin 23 receptor (*IL23R*), mRNA	NCBI Reference Sequence: NM_144701.2	1	chr1:67258271-67260319	11	GAAGGTGACTCCTGCTTCTCTCACTCTGC	ATAAGTAATGGGCCCAAGGTCACAATACAGGTAGC	3006
IL12RB2	Homo sapiens interleukin 12 receptor subunit beta 2 (*IL12RB2*), transcript variant 1, mRNA	NCBI Reference Sequence: NM_001559.2	1	chr1:67395283-67397253	16	TTGATCATTAACTGTCCTCTTTCCCTCCACAG	CCATGCCTAATTTTAAAACTTTTTGTAGAGAAGGGGTCTC	1526
IL6R	Homo sapiens interleukin 6 receptor (*IL6R*), transcript variant 1, mRNA	NCBI Reference Sequence: NM_000565.3	1	chr1:154464994-154469841	10	CATATTGCCCAGTGGTCACCTCACACTCC	TTCCCCTTCATGCCATTCCTTTAAAAGCATTATCCCTC	8024
IL1R2	Homo sapiens interleukin 1 receptor type 2 (*IL1R2*), transcript variant 1, mRNA	NCBI Reference Sequence: NM_004633.3	2	chr2:102027953-102028952	9	GTGATACTGACTTTATCAAATCTCTTATTTTTGCCTTGTGG	ATACTGGACTCAGACTCTAAGCCAACCTGC	5044
IL1R1	Homo sapiens interleukin 1 receptor type 1 (*IL1R1*), transcript variant 1, mRNA	NCBI Reference Sequence: NM_000877.3	2	chr2:102175409-102180452	12	GTTGGAACAAAGTTAAACCCTCAGCACTTGCC	GTTGGGTCTAATTCTCAGTTCACTAATACATTCCAATGTG	1000
IL1RL2	Homo sapiens interleukin 1 receptor like 2 (*IL1RL2*), mRNA	NCBI Reference Sequence: NM_003854.2	2	chr2:102239070-102240165	12	CTCACCTTACCCTCATCCTGTGCTATGAC	TCTGCCCCAAATAGCTGGTACATGTAACAACG	4096
IL1RL1	Homo sapiens interleukin 1 receptor like 1 (*IL1RL1*), transcript variant 1, mRNA	NCBI Reference Sequence: NM_016232.4	2	chr2:102343222-102346428	11	GTCTTCTCATGCAAATTTAATAAAGATTTGGAAGCAAGCAGG	TCATAGGTTCTATTATAGTATAAATAATATCCCAACTATGCCACAGG	2049
IL18R1	Homo sapiens interleukin 18 receptor 1 (*IL18R1*), transcript variant 1, mRNA	NCBI Reference Sequence: NM_003855.3	2	chr2:102396318-102399351	11	CTGGGTCTGGGAGAAAGTTCCTGATCCTG	TAACCCTGGCCTCCTTCTGCGATGAAGC	1517
IL18RAP	Homo sapiens interleukin 18 receptor accessory protein (*IL18RAP*), mRNA	NCBI Reference Sequence: NM_003853.3	2	chr2:102451705-102452728	12	CTGCCATATAAAAACTTAGGCCAGGCACAGTGG	CACATTTTGACATTCCTACCAGCCATCTGTGAGC	3049
IL5RA	Homo sapiens interleukin 5 receptor subunit alpha (*IL5RA*), transcript variant 1, mRNA	NCBI Reference Sequence: NM_000564.4	3	chr3:3070372-3066276	13	GATTTTCTGGGATTCTGTGTTAGGTGAGAGACAC	CACAGTCCATAGCTCTTGAGTCACTCTCAAATGC	1055
IL17RE	Homo sapiens interleukin 17 receptor E (*IL17RE*), transcript variant 5, mRNA	NCBI Reference Sequence: NM_153483.2	3	chr3:9915087-9916597	17	GATTTCAAAGAACCCAAAAGTTCTCTAAAAGACATCTAAGG	GAAAAATGAGAAAGGTACCCACCCCTCTTTAATTGC	4097
IL17RC	Homo sapiens interleukin 17 receptor C (*IL17RC*), transcript variant 1, mRNA	NCBI Reference Sequence: NM_153460.3	3	chr3:9932169-9934169	19	CTGATAGTTATTGCTCCTTTGAGCCGAAACCTGG	AGTTCAGGGTGGCCTTTGTGATAGTGTTACACC	4848
TGFBR2	Homo sapiens transforming growth factor beta receptor 2 (*TGFBR2*), transcript variant 2, mRNA	NCBI Reference Sequence: NM_003242.5	3	chr3:30691336-30694322	7	GTGAAGAAAGGGCTCATGAGCTGACGTGG	TTCCAAAGCAACTACCGTGTGGTTGCGTTAAAGC	4059
IL17RB	Homo sapiens interleukin 17 receptor B (*IL17RB*), mRNA	NCBI Reference Sequence: NM_018725.3	3	chr3:53864678-53866204	11	ATATACCACCAGATGAACTGACGGCAAGGTGG	TTCAGTGGACCACACCACGAAGGTAGTGG	1096
IL17RD	Homo sapiens interleukin 17 receptor D (*IL17RD*), transcript variant 2, mRNA	NCBI Reference Sequence: NM_017563.4	3	chr3:57097157-57089132	13	GAAATCCCAAAGACACAGACCCTACCCTCAG	CTTTACTTTCCTCATTAACTTGCTTTCACTGACTCTATGG	3078
IL1RAP	Homo sapiens interleukin 1 receptor accessory protein (*IL1RAP*), transcript variant 5, mRNA	NCBI Reference Sequence: NM_001167930.1	3	chr3:190630344-190629290	8	GCACTATGGTTTGGTCTGAGTACTGGCCAG	ACATGGGTGAACTTTGAAAACATGCTACATGAAAGAAGC	1036
IL1RAP	Homo sapiens interleukin 1 receptor accessory protein (*IL1RAP*), transcript variant 1, mRNA	NCBI Reference Sequence: NM_002182.3	3	chr3:190647945-190652003	12	GTTTGCACCTGCAAAGTAGGCATTAAAAGTAACAGG	ACTTTCTGTATACACATATAGCTAGGAAAATGCTGGCTG	3207
IL1RAP	Homo sapiens interleukin 1 receptor accessory protein (*IL1RAP*), transcript variant 6, mRNA	NCBI Reference Sequence: NM_001167931.1	3	chr3:190655786-190659884	12	CAAGTGTTTGTTCATTCCCTGTCCTCTGGAGG	TCACTAGGAAAGTCACTTAAGCTGGATAATATGGTCC	4099
KDR	Homo sapiens kinase insert domain receptor (*KDR*), mRNA	NCBI Reference Sequence: NM_002253.2	4	chr4:55080311-55078236	30	CTGAACTGAACATTTGATGGTGTGTCTCTCTGG	CTAATTTCCAGCCACTACTCTGCAGACTGC	4024
IL7R	Homo sapiens interleukin 7 receptor (*IL7R*), transcript variant 1, mRNA	NCBI Reference Sequence: NM_002185.3	5	chr5:35875914-35879937	8	TACTACGCAGCAGTAAAAGTGAATGGACTACAGC	CATAGCATGGTAAGAAAGACAGTTAATGTATACCTTGACG	1017
LIFR	Homo sapiens leukemia inhibitory factor receptor alpha (*LIFR*), transcript variant 2, mRNA	NCBI Reference Sequence: NM_002310.5	5	chr5:38482338-38474349	20	GTAAAAATTCTTATCCAGACCAATGGAAAATGGTGCATGC	AGGATTTAGTATTTCTTGTAAGGCAGACGTCCATAGG	5138
OSMR	Homo sapiens oncostatin M receptor (*OSMR*), transcript variant 3, mRNA	NCBI Reference Sequence: NM_001323504.1	5	chr5:38886703-38885687	7	TATAGCAACAAGGTCAGCAGGCCACCTTGC	CATGAGCAGCATCTGCTTCATCTCATATTACTGAGC	2987
OSMR	Homo sapiens oncostatin M receptor (*OSMR*), transcript variant 1, mRNA	NCBI Reference Sequence: NM_003999.2	5	chr5:38932598-38935699	18	CCACAGAGCCTAGACAGTGAGTTTCTCC	TTTCCCATCTCACCCCTCAGGATCTGGG	1102
IL31RA	Homo sapiens interleukin 31 receptor A (*IL31RA*), transcript variant 6, mRNA	NCBI Reference Sequence: NM_001297570.1	5	chr5:55908165-55909200	11	GAAAATGAGGCACATGCAGGTTAAGTGACTTGC	CAAAACCCTGTACTGGTCTGAGTATCCCAG	3062
IL31RA	Homo sapiens interleukin 31 receptor A (*IL31RA*), transcript variant 1, mRNA	NCBI Reference Sequence: NM_139017.5	5	chr5:55916572-55917619	15	CATTTCTCAGCCTACCACAGAAGCTGTTTGACC	GATTTCAGATTCTTGAAGACAAAATGCCTCTTGCCCTC	4127
IL31RA	Homo sapiens interleukin 31 receptor A (*IL31RA*), transcript variant 3, mRNA	NCBI Reference Sequence: NM_001242637.1	5	chr5:55922330-55923347	16	TGCTGTGTTTGCTCATTTACCCCAAATACTCATCC	AAGATGCTACGATAAATTGAAATTTATTACAACACAGTGAAATCAAGG	5056
IL6ST	Homo sapiens interleukin 6 signal transducer (*IL6ST*), transcript variant 1, mRNA	NCBI Reference Sequence: NM_002184.3	5	chr5:55942705-55934682	17	CTGCTATGCTGATTAACAACTGGGAGAGATACG	AAAACAACCACATGGAAATTATGTCTGATTCCTTCTTCTCC	2076
FLT4	Homo sapiens fms related tyrosine kinase 4 (*FLT4*), transcript variant 1, mRNA	NCBI Reference Sequence: NM_182925.4	5	chr5:180603428-180601458	30	AGAGTAGTAGAGACACCTGTTTACAAGCAGATTGG	CCAAAGAACAGGCTATATGTATGAACTTCCTAGCAC	7990
FLT4	Homo sapiens fms related tyrosine kinase 4 (*FLT4*), transcript variant 2, mRNA	NCBI Reference Sequence: NM_002020.4	5	chr5:180608857-180607795	30	TGTCAGAATCCTACTTTCCTAGAGCTCCTCAGG	TCATCTAAACTGAGTAATTACTAGATTTTCATTTCATTGAGGGAG	3102
IL11RA	Homo sapiens interleukin 11 receptor subunit alpha (*IL11RA*), transcript variant 3, mRNA	NCBI Reference Sequence: NM_001142784.2	9	chr9:34661246-34662253	13	TTCTTCTCTTACTAACACCACCTTCCCTGTCTTGG	CCATTTAGATTTATTACGGAAGCAAAAACAATAAAAGCAACACTCC	1971
TGFBR1	Homo sapiens transforming growth factor beta receptor 1 (*TGFBR1*), transcript variant 1, mRNA	NCBI Reference Sequence: NM_004612.3	9	chr9:99149110-99154247	9	TCCCTATGGCCTTTTCGCGCGTTCAAGG	ATTACTCATATTACCAAGGAATAACTGGTGGGCACAGG	1063
IL15RA	Homo sapiens interleukin 15 receptor subunit alpha (*IL15RA*), transcript variant 1, mRNA	NCBI Reference Sequence: NM_002189.3	10	chr10:5953381-5952322	7	TTTCAAGGTGTCTTGGATCAGTGCTAGGTCC	TGACCTCAGTCCCAACCCCTAATGCTGATC	1008
IL2RA	Homo sapiens interleukin 2 receptor subunit alpha (*IL2RA*), transcript variant 1, mRNA	NCBI Reference Sequence: NM_000417.2	10	chr10:6013145-6010097	8	TCTCAGGATGTTAGAGAATTTAACCAGCAGTGACC	AGTGTGGGCAGAAAGAGGGAGGATTCAGG	1486
IL10RA	Homo sapiens interleukin 10 receptor subunit alpha (*IL10RA*), transcript variant 1, mRNA	NCBI Reference Sequence: NM_001558.3	11	chr11:117998672-118001677	7	TAAAGAACAACAACAAAAACTAGCTGAGCCACTGC	TCTTCATGAATGTCAGAGAGATGGATACTGCTTGC	971
TNFRSF1A	Homo sapiens TNF receptor superfamily member 1A (*TNFRSF1A*), transcript variant 1, mRNA	NCBI Reference Sequence: NM_001065.3	12	chr12:6329793-6328692	10	AAGAAAGAAAGAAACAGGTTGAAGGTTGAGCAGG	GAGTTTACAATATAGGTCTTCACCATCCAGCTGC	1971
FLT1	Homo sapiens fms related tyrosine kinase 1 (*FLT1*), transcript variant 1, mRNA	NCBI Reference Sequence: NM_002019.4	13	chr13:28303719-28299593	30	CTAGGTTCTCTCCATCTCTGAGGACTAAGTGC	CTCTTTCTCCTCCTTTATGTGGAACATTTCTCCTTGG	1060
FLT1	Homo sapiens fms related tyrosine kinase 1 (*FLT1*), transcript variant 2, mRNA	NCBI Reference Sequence: NM_001159920.1	13	chr13:28390255-28385200	13	TCAGGCAGCCAAAGGTGGAATAGTAACATCTGG	CATGTTTTTCAGGGAGGCAGTGAAGCTGTGG	8028
IL4R	Homo sapiens interleukin 4 receptor (*IL4R*), transcript variant 1, mRNA	NCBI Reference Sequence: NM_000418.3	16	chr16:27362051-27365128	11	GATGGTTTACAGAGTGAAAGCCTGCTGTTTGC	TCGCTTTGTGACGTAGCCTGGTCTTGAGC	1527
IL21R	Homo sapiens interleukin 21 receptor (*IL21R*), transcript variant 1, mRNA	NCBI Reference Sequence: NM_021798.3	16	chr16:27448483-27452578	9	ATCCACTGGCTCTGACACTGTGCTGTGG	CAGAAGTCAGCCTCTTGTCTGAGGGTCTG	2001
EBI3	Homo sapiens Epstein-Barr virus induced 3 (*EBI3*), mRNA	NCBI Reference Sequence: NM_005755.2	19	chr19:4236625-4237723	5	GACCTCTCTGACTCAAGGTTTAACAATATCCATTGC	ATGGTAGTGCATGCTGTAATCCCAGTTAGAAGG	1024
IL27RA	Homo sapiens interleukin 27 receptor subunit alpha (*IL27RA*), mRNA	NCBI Reference Sequence: NM_004843.3	19	chr19:14051913-14053429	14	TCCTTCAGCATAGGGTCCCAGGTATGAGG	CAAACTTAGACTATATCCTGCTCGGGCAATGG	8026
IL12RB1	Homo sapiens interleukin 12 receptor subunit beta 1 (*IL12RB1*), transcript variant 1, mRNA	NCBI Reference Sequence: NM_005535.2	19	chr19:18060193-18058708	17	ACCCTATAGGCTAGGGCTGTTTTCGGTACC	CTCTGTTTTCCTCTCCTCTTGTAAGAATCACTGG	1511
IL12RB1	Homo sapiens interleukin 12 receptor subunit beta 1 (*IL12RB1*), transcript variant 2, mRNA	NCBI Reference Sequence: NM_153701.2	19	chr19:18071492-18070522	10	GTACATAATAAGTGCTAAGTAGGTGTTCATTATTATTGTTACC	TCCCTGGACTTACCATGCCTCAAGTTCCTG	3034
IL10RB	Homo sapiens interleukin 10 receptor subunit beta (*IL10RB*), mRNA	NCBI Reference Sequence: NM_000628.4	21	chr21:33296152-33297677	7	ATCTAGAACAGTGCCAGGCTCATAGCAGGTG	CTTCAGTTTTCCCTGCCTGGGAAATCGGAG	4019
IL17RA	Homo sapiens interleukin 17 receptor A (*IL17RA*), transcript variant 1, mRNA	NCBI Reference Sequence: NM_014339.6	22	chr22:17108127-17116154	13	TGTCAGCAATGCTCTCGTGGCTGTTCAAGG	AAGGAGCCTGGAATAGGAAGACAATGAGAGC	1018
IL2RB	Homo sapiens interleukin 2 receptor subunit beta (*IL2RB*), transcript variant 1, mRNA	NCBI Reference Sequence: NM_000878.4	22	chr22:37129177-37125159	10	CCTAACGAGTTTTTGGCTATGTCATATGTGACTCC	AAGGCTTCAGGGCTACCTTGTAGGGGAAG	1048

Supplementary Table 3. A list of 206 SNVs and 8 Indels in the gene loci of cytokine receptors detected in 30 IVIG-unresponsive KD patients (read depth >450, detected AAF >0.05).

gene	Chr	GCRh38 position	rs_ number	1KGP AAF	1KJPN AAF	KD AAF	Ref	Alt
TNFRSF1B	1	12207208	rs1061624	0.4565	0.4605	0.512	A	G
TNFRSF1B	1	12207235	rs3397	0.4091	0.3462	0.429	C	T
TNFRSF1B	1	12207407	rs5746065	0.1575	0.1391	0.14	C	A
TNFRSF1B	1	12207858	rs5746070	0.0256	0.07242991	0.101	C	G
TNFRSF1B	1	12207942	rs1061628	0.3592	0.2869	0.268	C	T
TNFRSF1B	1	12208442	rs1061631	0.1154	0.1479	0.152	G	A
IL23R	1	67259437	rs10889677	0.3926	0.6964	0.734	C	A
IL23R	1	67259964	-	-	-	0.054	G	A
IL12RB2	1	67395837	rs2229546	0.5551	0.4045	0.345	C	A
IL12RB2	1	67396783	rs1874396	0.8391	0.7706	0.688	G	T
IL12RB2	1	67396980	-	-	-	0.16	TA	-
IL6R	1	154465420	rs2229238	0.7969	0.8145	0.866	T	C
IL6R	1	154465608	rs7514452	0.7947	0.812	0.863	C	T
IL6R	1	154466404	rs4072391	0.7552	0.8113	0.859	T	C
IL6R	1	154467389	rs4379670	0.7959	0.8227	0.846	T	A
IL6R	1	154469622	-	-	-	0.05	A	G
IL1R2	2	102027999	rs3218984	0.2322	0.3363	0.341	T	C
IL1R1	2	102176300	rs3917318	0.3714	0.5897	0.6	A	G
IL1R1	2	102177073	rs3917324	0.1665	0.18691589	0.132	C	G
IL1R1	2	102177757	rs3732134	0.0581	0.1565	0.174	C	G
IL1R1	2	102177822	rs2110726	0.2554	0.3402	0.358	G	A
IL1R1	2	102178143	rs3732131	0.1821	0.2053	0.248	A	G
IL1R1	2	102180064	rs3917332	0.877	0.9958	1	A	T
IL1RL2	2	102239102	rs375155562	-	-	0.107	A	T
IL18R1	2	102396972	rs3771157	0.0687	0.0921	0.137	C	A
IL18R1	2	102397290	rs3732127	0.1887	0.1636	0.243	G	C
IL18R1	2	102397502	rs3732126	0.2344	0.1271028	0.414	A	C
IL18R1	2	102397503	rs3732125	0.2346	0.13364486	0.414	G	C
IL18R1	2	102397842	rs1135354	0.2552	0.3523	0.414	T	G
IL18R1	2	102398236	rs1568681	0.6871	0.5631	0.687	C	T
IL18R1	2	102399227	rs1420094	0.4321	0.2107	0.263	C	T
IL18RAP	2	102452327	rs7559479	0.6935	0.5586	0.724	G	A
IL5RA	3	3066399	rs340828	0.3496	0.2474	0.195	G	A
IL5RA	3	3066558	rs340829	0.3584	0.2577	0.272	T	G
IL5RA	3	3066596	rs55865625	0.0375	0.0864	0.118	C	G
IL5RA	3	3066953	rs340830	0.7354	0.4729	0.389	T	C
IL5RA	3	3067760	rs340831	0.6901	0.3952	0.389	C	T
IL5RA	3	3067948	rs340832	0.357	0.23411215	0.228	G	C
IL5RA	3	3068553	rs6768065	0.4143	0.2271028	0.281	A	T
IL5RA	3	3069774	rs340833	0.4247	0.2292	0.246	G	A
IL17RE	3	9915210	rs693802	0.3744	0.0684	0.104	C	T
IL17RE	3	9915771	rs1056286	0.3397	0.0688	0.117	C	T
IL17RE	3	9916214	rs8883	0.4257	0.06074766	0.125	G	A
IL17RC	3	9933125	rs76999397	0.0308	0.0412	0.077	G	A
IL17RC	3	9933563	rs183956	0.9872	0.9972	1	C	G
IL17RC	3	9933669	rs279551	0.9974	1	1	A	G
IL17RC	3	9933702	rs2270894	0.3013	0.52009346	0.299	C	G
TGFBR2	3	30691365	-	-	-	0.265	C	T
IL17RD	3	57089503	-	-	-	0.05	T	A
IL17RD	3	57089517	-	-	-	0.054	A	G
IL17RD	3	57090490	rs4681949	0.3986	0.1	0.074	C	T
IL17RD	3	57090563	rs4681950	0.3986	0.10981308	0.099	G	A
IL17RD	3	57091073	rs2035656	0.2095	0.3425	0.312	A	C
IL17RD	3	57091174	rs2035655	0.2632	0.3667	0.316	T	G
IL17RD	3	57091352	rs9311641	0.8594	0.528	0.493	C	T
IL17RD	3	57091396	rs17289035	0.2628	0.3673	0.335	G	A
IL17RD	3	57091699	rs11927968	0.222	0.0762	0.082	T	A
IL17RD	3	57091836	rs12486855	0.2063	0.3425	0.325	A	G
IL17RD	3	57092463	rs146995638	0.2039	-	0.235	-	C
IL17RD	3	57093963	rs2035653	0.7919	0.5262	0.499	G	A
IL17RD	3	57094183	rs11920551	0.1563	0.0594	0.053	G	A
IL17RD	3	57096616	rs56914717	0.2226	-	0.317	A	-
IL17RD	3	57096953	rs74494823	0.2061	0.33598131	0.303	T	G
IL1RAP	3	190629325	-	-	-	0.058	T	A
IL1RAP	3	190629602	rs7611887	0.9443	1	1	C	T
IL1RAP	3	190629734	rs1024946	0.6144	0.8001	0.821	G	A
IL1RAP	3	190629889	rs1024947	0.9441	1	1	C	G
IL1RAP	3	190629892	rs1024948	0.9441	1	1	C	T
IL1RAP	3	190630200	rs1058843	0.9447	1	1	A	C
IL1RAP	3	190649404	rs41392044	0.0294	0.0589	0.052	A	G
IL1RAP	3	190651042	rs41419651	0.0803	0.2986	0.179	C	T
IL1RAP	3	190651974	-	-	-	0.251	T	C
IL1RAP	3	190657779	rs80228668	0.0839	0.30046729	0.224	G	A
IL1RAP	3	190658675	rs79548924	0.1729	0.3014	0.19	G	A
IL1RAP	3	190658775	rs6763559	0.1092	0.30186916	0.197	C	T
IL1RAP	3	190658862	rs6763652	0.2372	0.3687	0.261	C	A
IL1RAP	3	190658954	rs6788031	0.7694	0.9486	0.958	A	T
IL1RAP	3	190658999	rs6788050	0.2372	0.3682	0.267	A	G
KDR	4	55079656	rs33978472	0.0733	-	0.07	A	-
KDR	4	55079914	rs4421048	0.9928	1	1	A	G
KDR	4	55080187	rs10006115	0.0725	0.0768	0.076	G	T
IL7R	5	35877724	rs9292617	0.6697	0.4766	0.477	A	T
IL7R	5	35877739	rs10491435	0.2372	0.0907	0.135	G	A
IL7R	5	35877812	rs10491434	0.2646	0.0911	0.113	A	G
IL7R	5	35878825	rs6451231	0.649	0.399	0.451	T	C
IL7R	5	35878993	rs6881270	0.2292	0.2226	0.193	C	T
IL7R	5	35879033	rs13167136	0.1284	0.1112	0.136	G	A
IL7R	5	35879493	rs700179	0.2646	0.0911	0.117	A	G
IL7R	5	35879501	rs1862632	0.1286	0.1112	0.132	A	G
LIFR	5	38474405	rs2731982	0.5909	0.707	0.785	T	C
LIFR	5	38475405	rs1046224	0.3728	0.6712	0.711	G	A
LIFR	5	38475925	rs3822425	0.3255	0.4341	0.572	T	C
LIFR	5	38477539	rs73077449	0.0655	0.2428	0.177	C	A

LIFR	5	38477761	rs34130318	0.3223	0.434	0.542	G	C
LIFR	5	38479725	rs60287085	0.0575	0.243	0.181	C	T
LIFR	5	38480590	rs3776424	0.0527	0.2301	0.159	T	C
LIFR	5	38480607	rs3776425	0.0659	0.243	0.188	C	T
LIFR	5	38481049	rs3822427	0.0577	0.2428	0.208	T	G
LIFR	5	38481233	rs1005017	0.729	0.7144	0.782	C	T
LIFR	5	38481271	rs3797156	0.0533	0.2299	0.184	C	T
OSMR	5	38885818	rs61166819	0.1554	0.0748	0.09	C	T
OSMR	5	38885916	rs664176	0.2476	0.0463	0.052	A	T
OSMR	5	38886321	rs74696378	0.0264	0.1341	0.145	A	G
IL31RA	5	55908195	-	-	-	0.07	G	T
IL31RA	5	55916618	rs16884641	0.2616	0.4034	0.356	T	G
IL31RA	5	55917195	rs161699	0.6965	0.8377	0.752	C	T
IL31RA	5	55923058	rs186560	0.3337	0.50841121	0.386	C	T
IL6ST	5	55936364	-	-	-	0.051	T	A
FLT4	5	180601509	rs72816988	0.0427	0.1083	0.144	G	A
FLT4	5	180601717	rs307822	0.6823	0.6088785	0.637	T	C
FLT4	5	180601743	rs2279622	0.1837	0.2001	0.095	C	T
FLT4	5	180602471	rs6877011	0.1717	0.393	0.215	C	G
FLT4	5	180602616	rs72816989	0.0661	0.1218	0.157	G	A
FLT4	5	180602879	rs72816990	0.0475	0.1215	0.149	G	A
FLT4	5	180608069	rs146943555	0.0525	0.16915888	0.157	G	A
FLT4	5	180608183	rs150066252	0.0351	-	0.08	AGT	-
FLT4	5	180608347	rs2387212	0.2115	0.24345794	0.173	C	T
FLT4	5	180608504	rs307833	0.8892	0.82943925	0.823	C	T
TGFBR1	9	99150189	rs334348	0.3319	0.5769	0.592	A	G
TGFBR1	9	99150733	rs11568811	0.0104	0.0351	0.088	G	C
TGFBR1	9	99152105	rs334349	0.3259	0.5752	0.645	G	A
TGFBR1	9	99152530	rs3739798	0.1266	0.4729	0.461	G	A
TGFBR1	9	99152566	rs117440593	0.007	0.035	0.067	T	C
TGFBR1	9	99152591	rs420549	0.1082	0.0678	0.111	G	C
TGFBR1	9	99153883	rs1590	0.3299	0.575	0.657	T	G
IL15RA	10	5952731	rs2296135	0.5667	0.4737	0.722	A	C
IL15RA	10	5953089	rs2229135	0.0875	0.098	0.091	C	T
IL2RA	10	6011323	rs12722604	0.094	0.3354	0.273	G	A
IL2RA	10	6011411	rs12244380	0.3548	0.1393	0.104	A	G
IL2RA	10	6011605	rs1570538	0.376	0.36495327	0.33	C	T
IL2RA	10	6013161	rs10508300	0.0725	0.0903	0.078	C	G
IL10RA	11	118001016	rs141609122	0.0032	0.0216	0.057	C	A
IL10RA	11	118001371	rs9610	0.5597	0.4874	0.448	G	A
FLT1	13	28299770	rs1830792	0.1647	0.2925	0.218	T	C
FLT1	13	28300125	rs9554312	0.0962	0.3036	0.362	A	G
FLT1	13	28301282	rs35779457	0.2352	-	0.341	-	TG
FLT1	13	28301297	rs55875014	0.2352	0.3335	0.398	G	C
FLT1	13	28301652	rs9554314	0.2388	0.3364	0.352	A	C
FLT1	13	28302077	rs7326277	0.261	0.3248	0.374	T	C
FLT1	13	28302136	rs56791288	0.2324	-	0.322	CTC	-
FLT1	13	28302820	rs3812867	0.014	0.0782	0.06	G	A
FLT1	13	28386331	rs7319123	0.9657	1	0.981	A	C
FLT1	13	28386429	rs3751396	0.0044	0.0481	0.137	A	G
FLT1	13	28386456	rs3751397	0.4141	0.5706	0.477	T	A
FLT1	13	28387316	rs193167358	0.0002	0.0121	0.054	A	G
FLT1	13	28388529	rs7337610	0.4856	0.5659	0.498	T	C
FLT1	13	28388549	rs17086617	0.2839	0.4144	0.485	T	C
FLT1	13	28388620	rs9551465	0.4	0.48878505	0.534	A	T
FLT1	13	28389539	rs2296284	0.2772	0.3785	0.448	G	A
FLT1	13	28389565	rs2296283	0.4016	0.572	0.488	G	A
FLT1	13	28390188	rs7983774	0.2792	0.3756	0.488	G	A
IL4R	16	27362237	rs3024676	0.2808	0.0496	0.061	C	A
IL4R	16	27362551	rs1805011	0.2364	0.0503	0.063	A	C
IL4R	16	27362594	rs2234898	0.2356	0.0504	0.059	G	T
IL4R	16	27362643	rs1805012	0.0881	0.0505	0.058	T	C
IL4R	16	27362651	rs2234900	0.2634	0.0497	0.056	T	C
IL4R	16	27362859	rs1805015	0.2017	0.0499	0.058	T	C
IL4R	16	27363079	rs1801275	0.3754	0.1201	0.129	A	G
IL4R	16	27363836	rs2074570	0.1196	0.0725	0.061	T	C
IL4R	16	27364008	rs563535954	0.0002	0.0095857	0.066	C	T
IL4R	16	27364221	rs1049631	0.4297	0.3324	0.361	G	A
IL4R	16	27364466	rs8832	0.4429	0.4135	0.444	A	G
IL4R	16	27364896	rs1029489	0.4559	0.4051	0.384	A	G
IL21R	16	27449576	rs2285452	0.2258	0.1341	0.1	G	A
IL21R	16	27449880	rs2239928	0.2254	0.1327	0.116	C	A
IL21R	16	27450370	rs2096	0.2262	0.1309	0.084	C	G
IL21R	16	27452127	rs3093390	0.225	0.1324	0.12	C	T
EBI3	19	4236999	rs4740	0.4485	0.37897196	0.367	G	A
EBI3	19	4237070	rs4905	0.4609	0.3771028	0.365	A	G
EBI3	19	4237428	rs6613	0.3842	0.38411215	0.374	A	T
IL12RB1	19	18059187	rs404733	0.4639	0.4453271	0.473	A	T
IL12RB1	19	18059574	rs3746190	0.1863	0.223	0.176	G	A
IL12RB1	19	18059847	rs12461312	0.1785	0.224	0.277	C	A
IL12RB1	19	18059870	rs17882555	0.1805	0.2286	0.239	G	A
IL12RB1	19	18060152	rs1870063	0.1805	0.2278	0.155	C	T
IL12RB1	19	18070759	rs845380	0.6585	0.65747664	0.724	A	G
IL12RB1	19	18070969	rs845381	0.099	0.20140187	0.125	G	A
IL12RB1	19	18071140	rs845382	0.0982	0.20046729	0.121	A	T
IL12RB1	19	18071189	-	-	-	0.092	G	A
IL10RB	21	33296442	rs3171425	0.5685	0.7973	0.697	A	G
IL10RB	21	33296671	rs8178562	0.1921	0.46869159	0.4	G	A
IL10RB	21	33297076	rs1058867	0.5651	0.7895	0.708	G	A
IL17RA	22	17108319	rs879577	0.2716	0.1007	0.17	C	T
IL17RA	22	17108407	rs2229151	0.1122	0.27196262	0.144	G	A
IL17RA	22	17108677	rs879575	0.1639	0.0589	0.056	C	T
IL17RA	22	17108904	rs12484684	0.113	0.2717	0.172	C	A
IL17RA	22	17109379	rs4819555	0.2426	0.0968	0.157	C	T
IL17RA	22	17109709	rs3804060	0.0304	0.0724	0.092	C	T

IL17RA	22	17109854	rs1468488	0.28	0.1044	0.174	T	C
IL17RA	22	17110199	rs2895332	0.731	0.7125	0.689	G	A
IL17RA	22	17110254	rs3179921	0.5397	0.42476636	0.463	C	T
IL17RA	22	17110807	rs882644	0.5555	0.42850467	0.491	G	A
IL17RA	22	17111920	rs12160599	0.0379	0.0933	0.136	A	G
IL17RA	22	17112164	rs4563337	0.0312	-	0.134	C	T
IL17RA	22	17112795	rs887796	0.8367	0.8041	0.803	G	A
IL17RA	22	17113582	rs9606617	0.1597	0.085	0.175	G	A
IL17RA	22	17113882	rs12484781	0.1627	0.2933	0.171	T	A
IL17RA	22	17113996	rs738035	0.4874	0.4248	0.541	T	C
IL17RA	22	17114025	rs738034	0.4864	0.4126	0.489	G	A
IL17RA	22	17114180	rs5992628	0.486	0.4144	0.499	T	G
IL17RA	22	17114856	rs1003945	0.0333	0.0927	0.136	C	T
IL17RA	22	17115025	rs3827278	0.1715	0.3246	0.343	C	A
IL17RA	22	17115039	rs3827279	0.1719	0.3232	0.325	G	C
IL17RA	22	17115076	rs1003944	0.0415	0.0791	0.097	C	T
IL17RA	22	17115090	rs1003943	0.0403	0.0925	0.15	C	T
IL17RA	22	17115288	rs4819962	0.6186	0.4248	0.594	T	C
IL17RA	22	17115339	rs1047929	0.1971	0.0846	0.144	T	C
IL17RA	22	17115432	rs1656	0.1715	0.3239	0.369	A	G
IL17RA	22	17115479	rs7289055	0.0333	0.0901	0.145	G	A
IL17RA	22	17115498	rs1654	0.8369	0.7991	0.831	G	C
IL2RB	22	37125509	rs58809963	0.0717	-	0.242	G	-
IL2RB	22	37125516	-	-	-	0.448	G	A
IL2RB	22	37127681	rs228941	0.3888	0.3629	0.294	C	G
IL2RB	22	37128579	rs228942	0.1803	0.2552	0.165	G	T

Supplementary Table 4. A list of 108 SNVs and 11 indels in the *IL4R* locus including the LD block with rs563535954 detected in 30 IVIG-unresponsive KD patients (read depth >1200, detected AAF >0.05).

gene	Chr	GCRh38 position	rs_ number	1KGP AAF	1KJPN AAF	KD AAF	Ref	Alt
IL4R	16	27309048	rs8057585	0.4545	0.6609	0.584	G	C
IL4R	16	27309294	rs397786699	-	-	0.942	-	G
IL4R	16	27310062	rs7205663	0.4139	-	0.585	C	T
IL4R	16	27310077	rs7205510	0.2953	-	0.554	A	G
IL4R	16	27310774	rs2057768	0.3387	0.6593	0.583	C	T
IL4R	16	27312083	rs2107356	0.3454	0.2671	0.301	C	T
IL4R	16	27313559	rs8060798	0.492	-	0.306	A	C
IL4R	16	27313560	rs8060938	0.4285	-	0.306	C	A
IL4R	16	27313700	rs12927172	0.5946	-	0.301	A	G
IL4R	16	27313702	rs12927543	0.9181	-	0.929	G	A
IL4R	16	27314947	rs7499292	0.5413	0.26588785	0.335	G	T
IL4R	16	27315521	rs8052962	0.5865	0.2648	0.337	T	G
IL4R	16	27315795	rs11645013	0.3844	0.2688	0.345	A	G
IL4R	16	27315893	rs8060025	0.5851	0.264	0.349	T	G
IL4R	16	27317797	rs7190969	0.9181	0.9237	0.942	G	A
IL4R	16	27318448	rs3052702	-	-	0.318	-	AA
IL4R	16	27319709	rs1076238	0.3383	0.2632	0.331	G	A
IL4R	16	27320573	rs6498011	0.5871	0.263	0.341	A	G
IL4R	16	27320653	rs6498012	0.5871	0.2657	0.339	C	G
IL4R	16	27321013	rs7187471	0.5871	0.261682243	0.332	A	G
IL4R	16	27321714	-	-	-	0.056	T	-
IL4R	16	27324278	rs11390919	0.4221	-	0.327	-	G
IL4R	16	27325106	rs1110470	0.4451	0.2647	0.34	G	A
IL4R	16	27325116	rs2040789	0.5054	0.2619	0.344	C	T
IL4R	16	27326401	rs117509107	0.0501	0.079439252	0.088	G	A
IL4R	16	27327703	rs71388093	0.3444	0.6557	0.579	T	C
IL4R	16	27327706	rs35004258	0.3568	0.6517	0.59	T	C
IL4R	16	27327824	rs9673499	0.3301	0.6503	0.609	T	C
IL4R	16	27328441	rs58653621	0.5323	0.648130841	0.575	T	G
IL4R	16	27329414	rs6498013	0.375	0.628037383	0.58	T	C
IL4R	16	27329738	rs4787948	0.351	0.6279	0.551	A	G
IL4R	16	27330232	rs7190472	0.406	0.623364486	0.598	T	C
IL4R	16	27331275	rs12925861	0.405	0.6273	0.574	T	A
IL4R	16	27331558	rs61373663	0.0861	0.1486	0.074	C	T
IL4R	16	27332158	rs2107355	0.3099	0.1486	0.527	A	G
IL4R	16	27332194	rs2107354	0.3918	0.477	0.572	T	C
IL4R	16	27332642	rs4787951	0.3051	0.477	0.514	T	C
IL4R	16	27332794	rs56943160	0.0865	0.1487	0.081	T	C
IL4R	16	27332878	rs35630199	0.1891	0.1481	0.072	T	G
IL4R	16	27333793	rs3916997	0.5254	0.6235	0.572	A	G
IL4R	16	27333800	rs7192673	0.0539	0.1487	0.054	T	C
IL4R	16	27334031	rs1981551	0.2704	0.470093458	0.483	C	T
IL4R	16	27335033	rs2283563	0.6112	0.6169	0.592	T	C
IL4R	16	27337309	rs12445247	0.3652	0.268224299	0.304	G	A
IL4R	16	27337847	rs3785356	0.263	0.471028037	0.507	C	T
IL4R	16	27338117	rs9940480	0.4625	0.620093458	0.61	T	C
IL4R	16	27338691	rs2382720	0.4994	0.6197	0.596	C	T
IL4R	16	27339366	rs3024530	0.4645	0.6214	0.475	A	G
IL4R	16	27340017	rs2057767	0.4495	0.621495327	0.564	A	G
IL4R	16	27340800	rs3024535	0.0565	0.1498	0.065	T	C
IL4R	16	27343210	rs3024548	0.5024	0.621	0.593	C	G
IL4R	16	27343412	rs752014969	-	-	0.443	-	TTTG
IL4R	16	27343683	rs3024552	0.4852	-	0.599	G	C
IL4R	16	27344041	rs3024556	0.3255	0.620093458	0.596	G	A
IL4R	16	27344882	rs1805010	0.456	0.6202	0.588	A	G
IL4R	16	27344903	rs144651842	0.0112	0.0684	0.093	G	A
IL4R	16	27345038	rs2074572	0.2917	0.4669	0.523	C	T
IL4R	16	27345077	rs2072130	0.3636	0.6002	0.614	C	T
IL4R	16	27345091	rs2074571	0.0567	0.1497	0.134	G	A
IL4R	16	27345346	rs3024560	0.4044	0.6192	0.59	T	G
IL4R	16	27346777	rs2301807	0.9521	0.9385	0.929	A	C
IL4R	16	27346811	rs3024575	0.3654	0.537	0.636	C	T
IL4R	16	27346882	rs3024577	0.5224	0.7136	0.708	A	G
IL4R	16	27347442	rs2283562	0.5236	0.544392523	0.576	T	C
IL4R	16	27347567	rs2239349	0.1639	0.1721	0.107	G	A
IL4R	16	27347627	rs2239348	0.6162	0.7131	0.709	A	G
IL4R	16	27347700	rs2239347	0.4661	0.5416	0.605	A	C
IL4R	16	27347740	rs2239346	0.4433	0.5395	0.593	A	T
IL4R	16	27348523	rs3024585	0.5112	0.7172	0.735	G	A
IL4R	16	27349306	rs3024589	0.5533	0.455140187	0.685	A	G
IL4R	16	27349307	rs3024590	0.5533	0.455140187	0.685	T	C
IL4R	16	27349846	rs2283561	0.4155	0.544392523	0.611	A	C
IL4R	16	27350041	rs2283560	0.0927	-	0.107	G	A
IL4R	16	27350472	rs2239345	0.1148	0.1701	0.11	C	T
IL4R	16	27352365	rs3024608	0.8806	0.9383	0.93	G	C
IL4R	16	27352837	rs3024610	0.3634	0.393	0.332	C	T
IL4R	16	27352879	rs3024611	0.7614	0.9383	0.934	A	C
IL4R	16	27352912	rs3024612	0.8205	0.9374	0.957	G	A
IL4R	16	27352932	rs3024613	0.3624	0.3939	0.331	C	T
IL4R	16	27353024	rs3024614	0.8205	0.9383	0.929	G	A
IL4R	16	27353485	rs3024619	0.3572	0.5444	0.596	G	A
IL4R	16	27353597	rs3024620	0.8806	0.9382	0.93	G	C
IL4R	16	27354132	rs3024622	0.3564	0.5449	0.589	C	G
IL4R	16	27354293	rs3024624	0.363	0.3935	0.306	A	G
IL4R	16	27354709	rs3024629	0.883	0.9378	0.933	A	G
IL4R	16	27356013	rs4787423	0.8081	0.942	0.925	C	T
IL4R	16	27356505	rs3024644	0.7887	0.9402	0.919	C	G
IL4R	16	27356651	rs3024647	0.7881	0.9397	0.934	G	A
IL4R	16	27356678	rs3024648	0.8666	0.9397	0.918	C	T
IL4R	16	27357466	-	-	-	0.888	-	T
IL4R	16	27357469	rs2891057	0.7402	0.900934579	0.882	A	T
IL4R	16	27357512	rs2891058	0.7039	0.937850467	0.915	G	A
IL4R	16	27358181	rs3024655	0.7678	0.936915888	0.914	A	G
IL4R	16	27359133	rs3024658	0.8431	0.9364	0.908	A	G
IL4R	16	27359613	rs138640688	0.7236	-	0.885	TGG	-
IL4R	16	27359837	rs3024660	0.7236	0.9377	0.881	C	T
IL4R	16	27360076	rs3024662	0.3844	0.377570093	0.314	G	A
IL4R	16	27360103	rs3024664	0.8203	0.935981308	0.922	T	C
IL4R	16	27360218	rs3024665	0.77	0.935981308	0.918	C	T
IL4R	16	27360250	rs3024666	0.7232	0.935514019	0.915	G	A
IL4R	16	27360263	rs3024667	0.7806	0.935514019	0.925	T	C
IL4R	16	27360628	rs3024668	0.8129	0.9386	0.914	A	G
IL4R	16	27361451	rs538765536	0.00019	0.0271	0.132	A	C

IL4R	16	27362237	rs3024676	0.2808	0.0496	0.057	C	A
IL4R	16	27362551	rs1805011	0.2364	0.0563	0.054	A	C
IL4R	16	27362651	rs2234900	0.2634	0.0497	0.05	T	C
IL4R	16	27362859	rs1805015	0.2017	0.0499	0.057	T	C
IL4R	16	27363079	rs1801275	0.3754	0.1201	0.101	A	G
IL4R	16	27364008	rs563535954	0.0002	0.0095857	0.076	C	T
IL4R	16	27364221	rs1049631	0.4297	0.3324	0.389	G	A
IL4R	16	27364466	rs8832	0.4429	0.4135	0.467	A	G
IL4R	16	27364896	rs1029489	0.4559	0.4651	0.352	A	G
IL4R	16	27365316	rs2382721	0.3474	0.5964	0.601	A	G
IL4R	16	27365589	rs3024685	0.5473	0.5969	0.624	T	C
IL4R	16	27365690	rs35146330	-	-	0.642	-	C
IL4R	16	27365843	rs72064218	0.5471	-	0.602	TGA	-
IL4R	16	27366463	rs368508458	-	-	0.929	A	-
IL4R	16	27366471	rs572510621	0.0008	-	0.071	-	AC
IL4R	16	27366732	rs12102586	0.1308	-	0.058	C	T

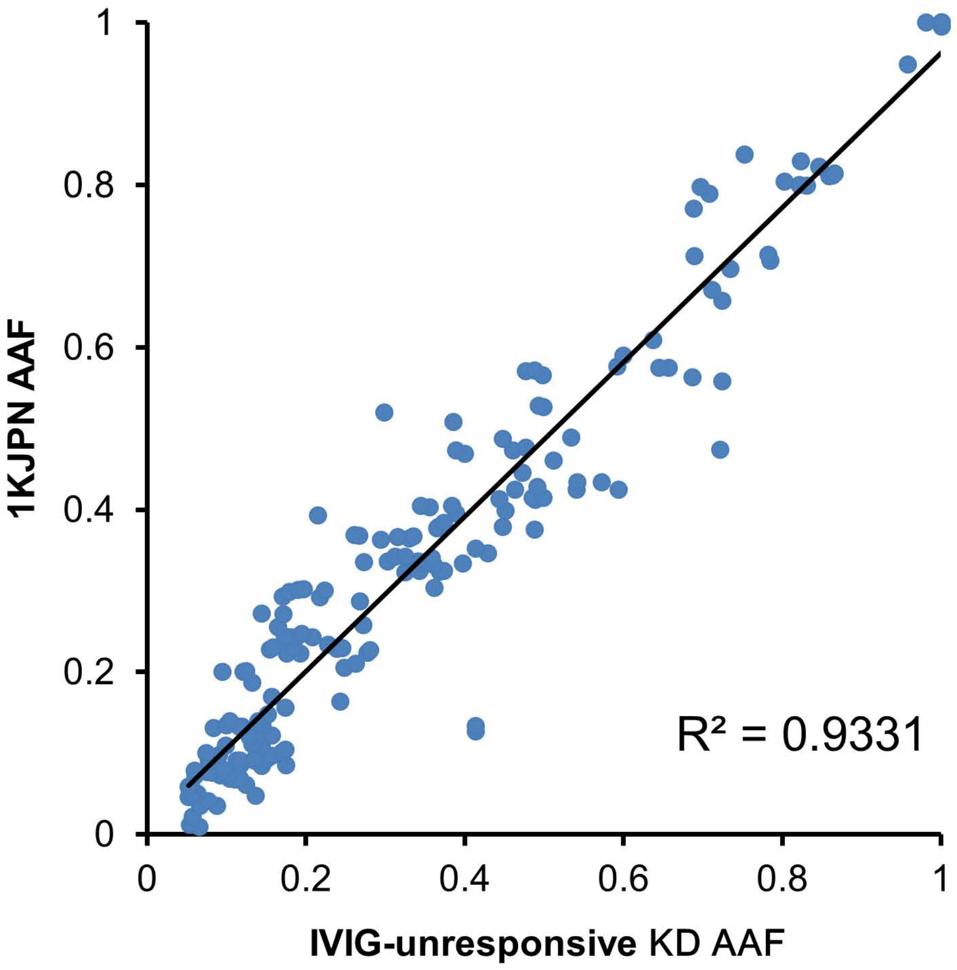

Supplementary Figure 1. Pairwise correlation between AAFs of SNVs, estimated via pooled genome sequencing of IVIG-unresponsive KD patients, in the gene loci of cytokine receptors and AAFs found in 1KJPN. The square of Pearson's correlation coefficient, R^2=0.9331.

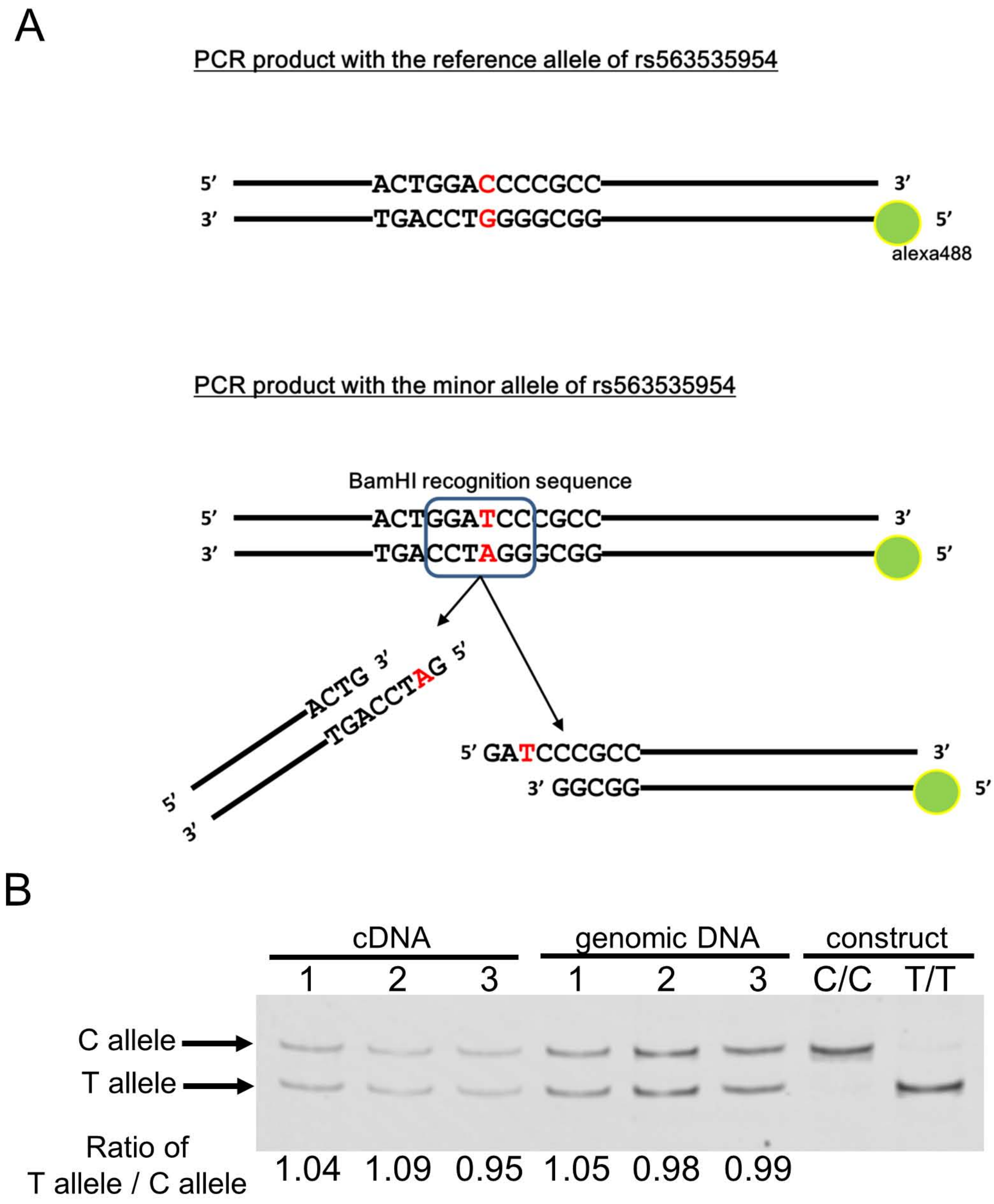

Supplementary Figure 2. Allele-specific transcript quantification of *IL4R* in PBMCs from IVIG-unresponsive KD patients with heterozygous rs563535954. (A) Schematic diagram of the separation-detection method of *IL4R* transcripts derived from an allele with or without the minor allele of rs563535954 based on PCR-restriction fragment length polymorphism (PCR-RFLP). PCR products derived from the minor allele of rs563535954 (T) and the counterpart allele (C) can be distinguished by *Bam*HI digestion. Homoduplex products are labelled with Alexa-488. (B) Comparison of the abundance of *IL4R* transcripts between those with or without the minor allele of rs563535954 in PHA stimulated PBMCs from KD patients with heterozygous rs563535954. Numerals indicate individual patients. PCR products amplified from genomic DNA indicate an equal ratio between the alleles. PCR products amplified from constructs were used as controls for specific digestion (C/C) and complete digestion (T/T) by *Bam*HI. The fluorescence ratio of Alexa-488 labelled fragments derived from the minor allele of rs563535954 (T) and the counter allele (C) are indicated at each line. Image presented in this panel was cropped to remove surrounding white space.

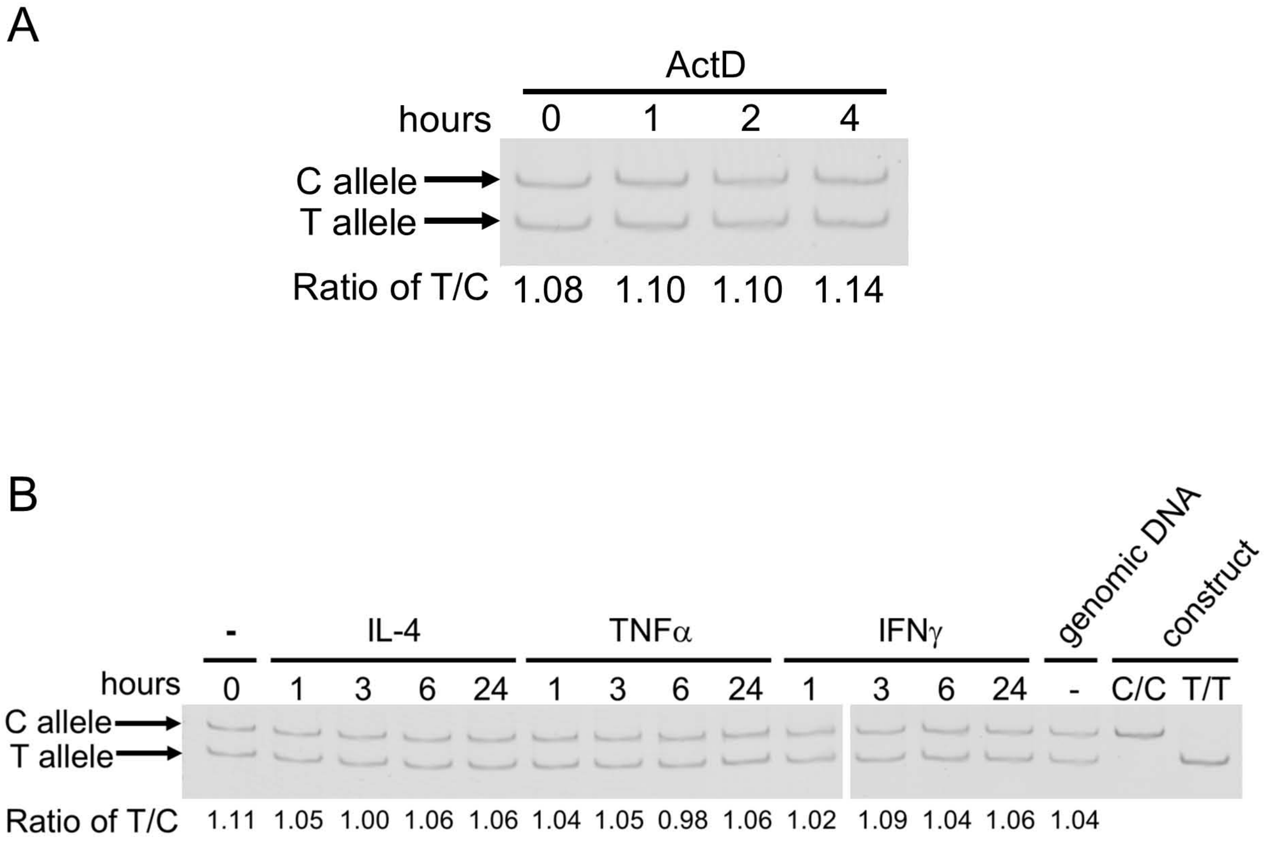

Supplementary Figure 3. Allele-specific transcript quantification of *IL4R* in B-LCLs derived from individuals with heterozygous rs563535954. B-LCLs derived from individuals with heterozygous rs563535954 were incubated with or without Actinomycin D (A), IL-4, TNFα or IFNγ (B) for the indicated times. The abundance of *IL4R* transcripts between those with or without the minor allele of rs563535954 was compared by a PCR-RFLP-based method under each of the conditions. PCR products amplified from genomic DNA indicate an equal ratio between the alleles. PCR products amplified from constructs were used as controls for specific digestion (C/C) and complete digestion (T/T) by *Bam*HI. The T/C ratio represents the mean values of the fluorescence ratio of Alexa-488 labelled fragments derived from the minor allele of rs563535954 (T) and the counter allele (C) obtained from three B-LCL cell lines. Images presented in this panel were cropped to remove surrounding white space.

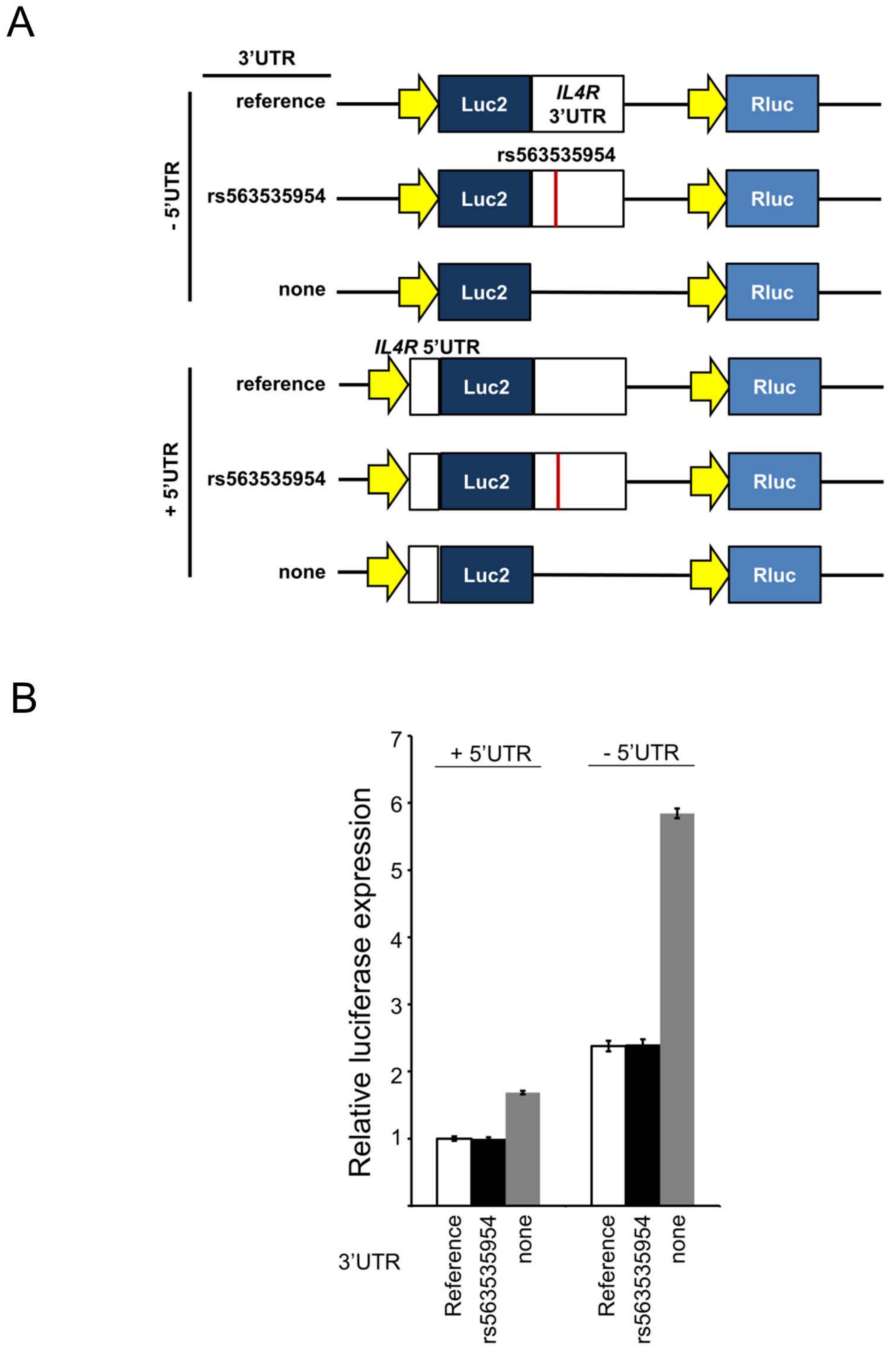

Supplementary Figure 4. Effect of the minor allele of rs563535954 on translational efficiency. (A) Dual luciferase expression constructs used in this assay are shown. (B) HEK293T cells were transfected with dual luciferase expression constructs (pmirGLO) as indicated, and the cells were incubated for 24 hours and then harvested for dual luciferase assay. Error bars represent the s.d. from five independent replicates.

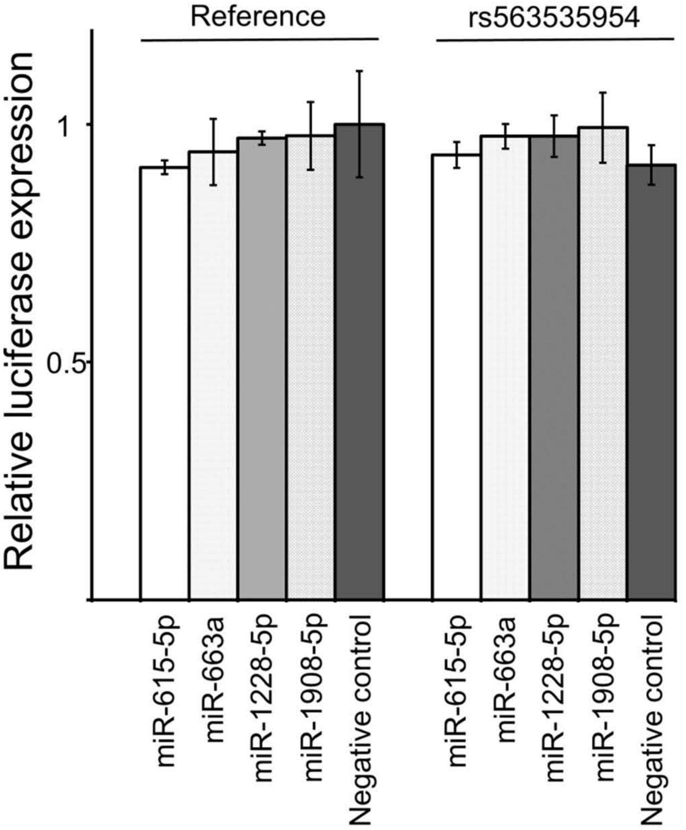

Supplementary Figure 5. The effect of miRNA mimics on the activity of luciferase expressed from a reporter plasmid containing the 3'-UTR of IL-4Rα. HeLa cells were co-transfected with a pmiRGLO luciferase reporter plasmid containing the 3'-UTR of IL-4Rα, with or without the minor allele of rs563535954 and miRNA mimics, as indicated. Cells were harvested for assessment via dual luciferase assay after 24 hours of incubation. Error bars represent the s.d. from four independent replicates.

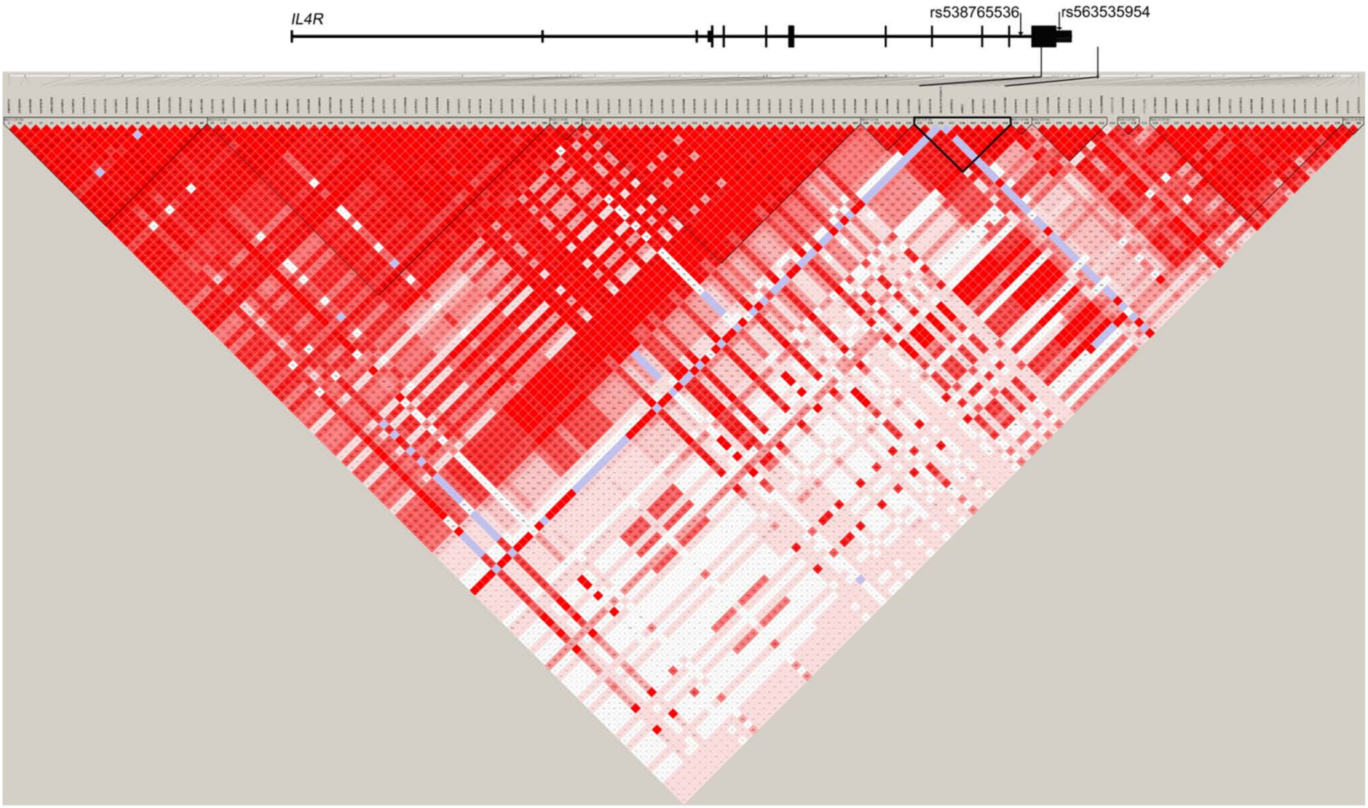

Supplementary Figure 6. Linkage disequilibrium (LD) plot around the *IL4R* gene locus and *IL4R* gene structure. The LD block is based on the identified variants in 1KJPN (AAF >0.05). Haplotype block containing rs563535954 is surrounded by a bold line. Location of rs563535954 and rs538765536 are shown on the schematic diagram of the *IL4R* gene.

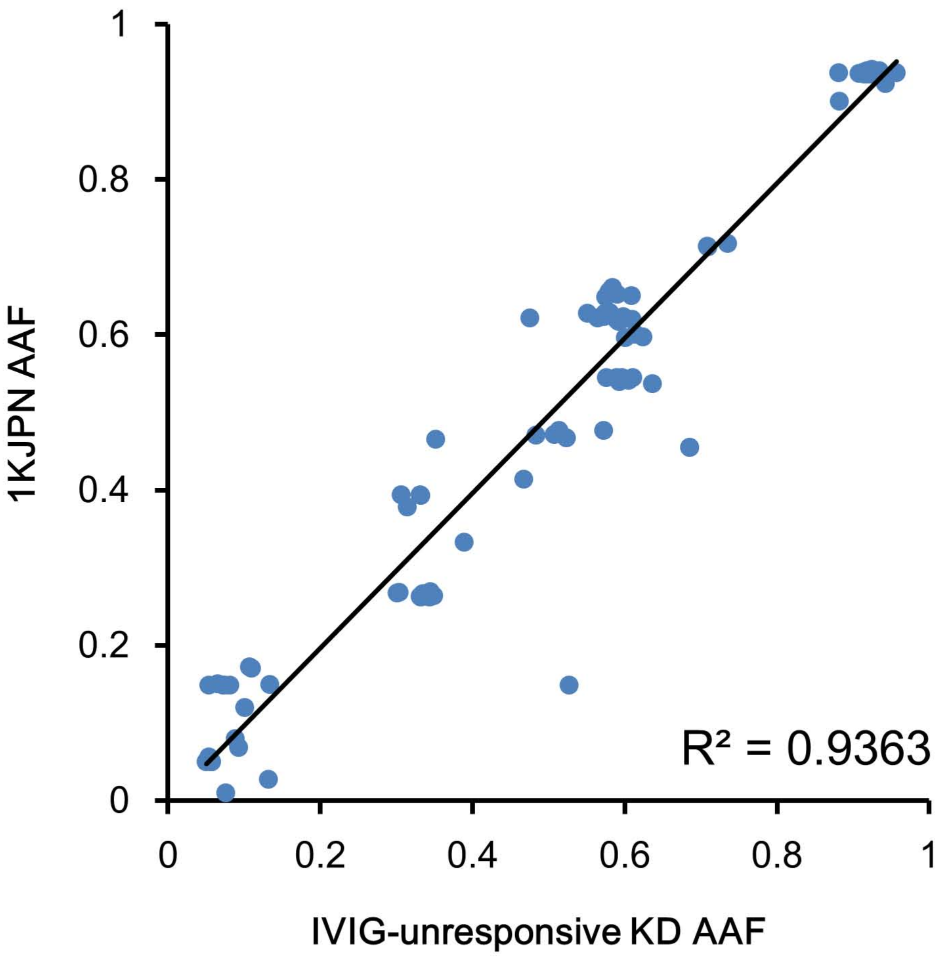

Supplementary Figure 7. Pairwise correlation between the AAFs of SNVs, estimated via the pooled genome sequencing of IVIG-unresponsive KD patients, in the *IL4R* gene locus including the LD block with rs563535954 and AAFs of that found in 1KJPN. The square of Pearson's correlation coefficient, R^2=0.9363.
