# Supplementary figures and images for: A low-frequency IL4R locus variant in Japanese patients with intravenous immunoglobulin therapy-unresponsive Kawasaki disease
Source: Pediatr Rheumatol Online J. 2019 Jul 3;17:34. doi: 10.1186/s12969-019-0337-2 (PMC6610867; doi:10.1186/s12969-019-0337-2)

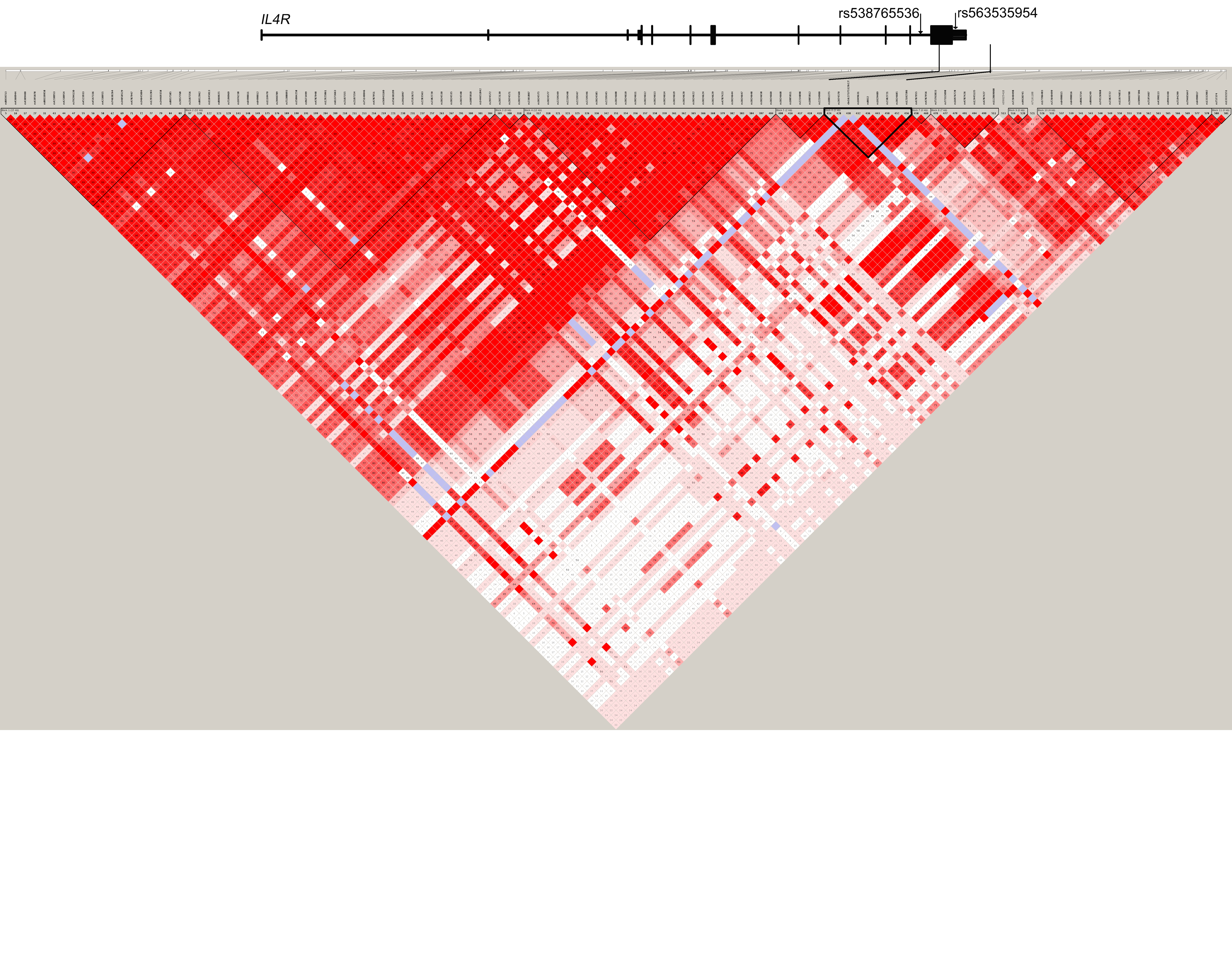

Supplement: Supplementary file 2 — High-resolution image of Additional file 1: Figure S6. (TIF 3930 kb) [file 12969_2019_337_MOESM2_ESM.tif]
